# Predicting the Fission Yeast Protein Interaction Network

**DOI:** 10.1534/g3.111.001560

**Published:** 2012-04-01

**Authors:** Vera Pancaldi, Ömer S. Saraç, Charalampos Rallis, Janel R. McLean, Martin Převorovský, Kathleen Gould, Andreas Beyer, Jürg Bähler

**Affiliations:** * Department of Genetics, Evolution, and Environment and; † UCL Cancer Institute, University College London, London WC1E 6BT, United Kingdom; ‡ Cellular Networks and Systems Biology, Biotechnology Center, Dresden University of Technology (TU Dresden), Dresden 01307, Germany, and; § Howard Hughes Medical Institute; ** Department of Cell and Developmental Biology, Vanderbilt University School of Medicine, Nashville, Tennessee 37232

**Keywords:** Cbf11, TOR, Mak1/2/3, support vector machine, random forest

## Abstract

A systems-level understanding of biological processes and information flow requires the mapping of cellular component interactions, among which protein–protein interactions are particularly important. Fission yeast (*Schizosaccharomyces pombe*) is a valuable model organism for which no systematic protein-interaction data are available. We exploited gene and protein properties, global genome regulation datasets, and conservation of interactions between budding and fission yeast to predict fission yeast protein interactions *in silico*. We have extensively tested our method in three ways: first, by predicting with 70–80% accuracy a selected high-confidence test set; second, by recapitulating interactions between members of the well-characterized SAGA co-activator complex; and third, by verifying predicted interactions of the Cbf11 transcription factor using mass spectrometry of TAP-purified protein complexes. Given the importance of the pathway in cell physiology and human disease, we explore the predicted sub-networks centered on the Tor1/2 kinases. Moreover, we predict the histidine kinases Mak1/2/3 to be vital hubs in the fission yeast stress response network, and we suggest interactors of argonaute 1, the principal component of the siRNA-mediated gene silencing pathway, lost in budding yeast but preserved in *S. pombe*. Of the new high-quality interactions that were discovered after we started this work, 73% were found in our predictions. Even though any predicted interactome is imperfect, the protein network presented here can provide a valuable basis to explore biological processes and to guide wet-lab experiments in fission yeast and beyond. Our predicted protein interactions are freely available through PInt, an online resource on our website (www.bahlerlab.info/PInt).

Progress in experimental techniques has uncovered highly interconnected protein networks. Protein interactions not only hold together protein complexes, the operational units within cells, but also mediate cellular information flow along signaling pathways and networks. Acquiring a global knowledge of protein interconnections is important for understanding biological processes and their disruption in disease.

Several high-throughput approaches for experimental detection of physical protein interactions have been applied, such as affinity capture followed by mass spectrometry (MS) (Gavin *et al.* 2002; Krogan *et al.* 2006), yeast two-hybrid (Y2H) (Ito *et al.* 2001; Uetz *et al.* 2000), and kinase interaction assays (Breitkreutz *et al.* 2010). These techniques have now provided protein interactomes for several organisms (Formstecher *et al.* 2005; Li *et al.* 2004; Rual *et al.* 2005; Stelzl *et al.* 2005). Many additional protein interactions are known from various small-scale experiments, such as co-immunoprecipitations or affinity capture, which provide us with data for species in which large-scale studies are not yet available. The budding yeast *Saccharomyces cerevisiae* is the model organism in which the largest portion of protein interactions has been studied experimentally, covering ∼56,363 unique interactions among 5786 proteins (Stark *et al.* 2011). Typical overlaps between different Y2H screens are around 20% (Yu *et al.* 2008), and similar for TAP-MS (Gavin *et al.* 2011). In addition, the experimental exploration of the interactome is prone to some bias, because better-studied proteins are more likely to be used as baits in experiments (Yu *et al.* 2008) as well as because of technical biases. For example, a network distance estimation method applied to interaction datasets obtained with different techniques on different species produced clusters based on the interaction detection method rather than on the species (Fernandes *et al.* 2010). A recent report has developed a confidence score for each experimentally verified interaction (Braun *et al.* 2009), the use of which could potentially mitigate some of these biases. However, paying attention to the quality of the data is not common practice. We are still far from a complete and reliable picture of the protein interactome, even for the intensely studied budding yeast.

The fission yeast *Schizosaccharomyces pombe* is only remotely related to budding yeast and provides a powerful complementary model system. However, no high-throughput protein interaction screens have been performed in fission yeast, and relatively few small-scale experimental data on protein interactions are available: a total of 2719 unique interactions involving 1468 proteins have been reported so far (Stark *et al.* 2011). Orthology relationships have been used for mapping interactions measured in one species onto other species (Jensen *et al.* 2009). The considerable level of conservation at the protein complex level suggests that such inter-species mapping could successfully update our knowledge about protein binding in fission yeast based on data in budding yeast. However, such mapping will be restricted to network modules existing in both species. Hence, it is unavoidable that modules within this network that are different between the two organisms will remain incorrectly or incompletely characterized (Roguev *et al.* 2004).

Assaying all possible protein interactions experimentally in several species, in multiple repeats and under different conditions remains an enormously expensive and time-consuming challenge. Hence, there is a need for developing approaches that can predict physical protein interactions based on some pre-existing experimental data. Pitre *et al.* (2008) have reviewed the main types of prediction approaches that show promising results, and Qi *et al.* (2006) conducted a comparison of the available methods. Different approaches have used protein sequences (Shen *et al.* 2007), functions (Ben-Hur and Noble 2005) or three-dimensional structures (Aloy and Russell 2002; Burger and Van Nimwegen 2008; De Bodt *et al.* 2009; Jansen *et al.* 2003; Singh *et al.* 2010; Wang *et al.* 2009a). In this article, we suggest a supervised machine learning approach for the prediction of physical protein-protein interactions based on a collection of more than 100 different protein and gene features, using negative sets that preserve the degree of each protein as in the positive set. We present the first large-scale protein interaction network for fission yeast. Contrary to the existing fission yeast interactome available through STRING (Szklarczyk *et al.* 2011), we only consider physical protein interactions, and we do not assume all interactions in budding yeast to be conserved. We present experimental verification of some of our predictions, and we make the whole dataset available for further exploration on our website (www.bahlerlab.info/PInt).

## MATERIALS AND METHODS

### Assembling the features

The machine learning approach suggested requires positive and negative training sets of interactions as well as a vector of features for each pair of proteins. The feature vector was assembled by concatenating the features of the first and second protein as well as the corresponding pair features. However, to ensure consistency in defining symmetric interactions (if A interacts with B, it follows that B interacts with A), we ordered the features such that the smaller value always came first. In a previous version, the two symmetric interactions were both included, but this slowed down the training procedure and did not lead to better results.

### Assembling the training sets

We then used these features to classify any two proteins into either interacting or not interacting pairs. The starting point was a positive training set (list of known interacting pairs) and a negative training set (list of pairs believed not to interact).

### Positive training set

In recent years, numerous interaction databases have attempted to summarize the knowledge on protein interactions in different species. We used data from BioGRID (Stark *et al.* 2011), which aims to integrate the information coming from different sources with a systematic evidence code. The main types of documented physical interactions are subdivided into two classes: large-scale experiments, usually less accurate but unbiased, and small-scale experiments, probably of higher quality but with much less coverage and obvious biases dictated by the higher biological interest of certain proteins. Of all the types of small-scale experiments included in BioGRID, we only considered the ones that aim to prove a physical interaction, thus including co-immunoprecipitation, capture experiments of different types, and complex reconstitution, but excluding co-localization. As far as the large-scale experiments are concerned, the most common experiments are affinity capture-MS and Y2H. Some of the interactions in databases are of dubious reproducibility, and especially, Y2H and capture methods are known to have different technical biases. We thus took a strict approach, including these interactions in our training sets only if they were verified by both Y2H and a capture technique, either large-scale, like affinity capture-MS, or small-scale, like affinity capture. This filtering reduced our training sets considerably, but it increased the quality of the training data. We chose to consider only orthologs from budding yeast as we assumed that complexes in the two species are highly conserved, which was shown to be particularly true between the two yeasts (Dixon *et al.* 2008; Roguev *et al.* 2008). Only unique orthologs were considered, and orthology mapping was performed with a list of one-to-one orthologs curated by an expert (Wood 2006). A previous approach mapping interactions from more species led to worse results. The resulting high-quality positive training set consisted of 1097 interactions among 660 proteins.

### Negative training set

A common strategy to generate pairs of non-interacting proteins is to choose proteins with different GO annotation or different localization. However, Ben-Hur and Noble (2006) have shown that this introduces biases in the sets that can confound the algorithm, as there could be differences in proteins that are found in different cell compartments. Moreover, in species with few known interactions, we do not want to introduce any bias in the type of proteins for which we predict interactions. The commonly used alternative is to create random protein pairs, on the assumption that it is highly unlikely that randomly chosen pairs physically interact, given the space of possible interactions is of the order of the square of the number of proteins. However, a recent report has shown that the resulting randomized negative sets are not comparable to the positive sets, unless the randomization preserves the number of connections for each protein (Yu *et al.* 2010). For this reason, the construction of a degree-balanced negative set, which does not give more or less importance to the interactions involving different proteins, has been advocated (Yu *et al.* 2010). Hence, our negative set was constructed by shuffling the links between the proteins in the positive set while keeping the degree fixed, *i.e.* the number of connections for each protein. We also removed from the pool of possible negative pairs any interactions that have been experimentally shown at least once with any method in either of the two yeasts. The negative sets were generated using BRS-nonint (Yu *et al.* 2010).

### Support Vector Machines and Random Forest

Using the features and training sets described above, we trained a support vector machine (SVM) and a random forest (RF). The features for all protein pairs were concatenated in feature vectors for the positive and negative training sets, and only pairs for which none of the features were missing were used for the classification. Training and testing of the SVM was performed using a radial basis function kernel with the e1071 package for R. Optimal parameters were chosen using the tune function in a cross-validation framework. The random forests were built using the package randomForest, and the cforest function from the party package was used for estimating the feature importance (Strobl *et al.* 2008).

### Classification performance measures and ROC curves

The performance of the classification was estimated using standard measures. Once a specific threshold is chosen for the interaction score, we can define the following: true positives (TP) as the number of predicted interactions which are true interactions; true negatives (TN) as the number of false interactions correctly not predicted; false positives (FP) as the number of false interactions incorrectly predicted; and false negatives (FN) as the number of true interactions not predicted. Using these measures, we can define the following: specificity = TN/(TN+FP); sensitivity = TP/(TP+FN); accuracy = (TP+TN)/(TP+TN+FP+FN); and false-positive rate (FPR) = FP/N = FP/(FP+TN). Moreover, we used Receiver Operator Characteristic curves (ROC), Areas Under the Curve (AUC), areas under the partial curve (AUC50), and precision-recall curves as a threshold-independent measure of the performance, using the ROCR package for R. The main purpose of providing these measures for our classification was to be able to compare the RF and SVM methods.

### Test sets

We constructed a positive test set by collecting high-confidence protein interactions in fission yeast. We found 204 pairs of proteins that were shown to interact both by Y2H and a different capture experiment in fission yeast (BioGRID). Any interactions involving proteins that appear in this high-confidence fission yeast interactome were eliminated from our training sets. We then proceeded to build negative test sets.

The first negative test set was constructed making random pairs of the proteins from the positive set, eliminating all interactions documented at least once in budding or fission yeast and preserving the degree of each protein. This ensures that the positive and negative test sets contain the same proteins (none of them present in the training set) and have the same topological features.

In a more realistic scenario, there should be a strong imbalance between the number of positive and negative interactions, so we proceeded to also generate a negative set to reproduce this bias. To construct this negative test set, we made random pairs of the proteins known to interact in budding or fission yeast paired at random, maintaining the same degree for each protein. This degree-balanced negative set comprises 32,482 pairs among ∼4500 genes, which, combined with the 204 positives, leads to a ratio between positives and total pairs of ∼0.006, the same as what we expect for the real interactome [12.5 million possible pairs; an upper estimate of between 60,000 and 80,000 true interactions (Hart *et al.* 2006)].

### Prediction of SAGA, a large co-activator complex

The Spt-Ada-Gcn5 acetyltransferase (SAGA) complex is involved in regulating RNA polymerase II transcription through chromatin remodeling and histone modification. It is one of the largest and best-annotated protein complexes in budding and fission yeast (Helmlinger *et al.* 2008, 2011; Koutelou *et al.* 2010) and, as such, suitable for testing our method. To test our method using the SAGA complex, we constructed a test set of all possible pairs between the fission yeast SAGA components and an equal number of control proteins picked at random. The positive and degree-balanced negative training sets were constructed so that they would not include any of the test proteins. We then predicted all possible interactions among the SAGA proteins and the control proteins. We also predicted interactions for the SAGA proteins with all fission yeast proteins and compared the overlap between the predicted interactions and the real list of SAGA interactions.

### Cbf11 predictions

As there were no interactions for Cbf11 in the training set, the original training pairs were used to make predictions about Cbf11 interactions with all the 4920 fission yeast proteins for which data were available. We predicted interactions using both SVM and RF, applying a score threshold of 0.5 to separate positives from negatives. We then tested these predictions against the results of an affinity capture-MS experiment that used TAP-tagged Cbf11 as bait for the affinity purification. We also tested the enrichment of our predicted interaction partners for experimentally verified interactors and compared this overlap with expected overlaps of randomized lists of interactions.

The predicted and experimentally verified interactors were checked against a list of known complexes in fission yeast to examine the coverage of the different complexes within the experimentally verified associated proteins. Gene Ontology (GO) enrichment analysis was performed using GO TermFinder (Boyle *et al.* 2004).

### Protein purification and mass spectrometry

Fission yeast strain (JB795) expressing a C-terminally TAP-tagged version of Cbf11 from its endogenous chromosomal locus was grown to mid-exponential phase in 4× concentrated YES medium (Formedium), washed twice with water, and snap-frozen in liquid nitrogen. Cells were broken in the RM 200 mortar (Retch), and lysates were clarified by ultracentrifugation in the Sorvall Discovery 90SE ultracentrifuge (Sorvall T-647.5 rotor) at 35,000 rpm for 90 min. Due to low abundance of the bait protein, only the first purification step of the standard TAP protocol (Rigaut *et al.* 1999) was employed. Briefly, the tagged protein with its interactors were captured on IgG-coated magnetic beads (1:1 pan mouse IgG and M-280 sheep anti-rabbit IgG dynalbeads, Invitrogen), washed three times with NP-40 lysis buffer, three times with TEV cleavage buffer, and eluted in 2× 500 µl elution buffer (0.5 M NH_4_OH, 0.5 mM EDTA). The purified proteins were then TCA-precipitated and digested with trypsin (Promega). The resultant peptides were subjected to two-dimensional LC-MS/MS analysis on a Thermo LTQ as previously detailed (Mcdonald *et al.* 2002; Roberts-Galbraith *et al.* 2009). Thermo RAW files were converted to MZML files using Scansifter (software developed at the Vanderbilt University Medical Center). Spectra with fewer than 20 peaks were excluded from our analysis. The *S. pombe* database (http://www.sanger.ac.uk, October 2009) was searched with the MyriMatch algorithm (Tabb *et al.* 2007) v1.6.33 on a high-performance computing cluster (Advanced Computing Center for Research and Education at Vanderbilt University). We added contaminant proteins (*e.g.*, keratin, IgG) to the complete *S. pombe* database and reversed and concatenated all sequences to allow estimation of FDRs (10,186 entries). MyriMatch parameters were as follows: strict tryptic cleavage; modification of methionine (oxidation, dynamic modification, +16 Da), S/T/Y (phosphorylation, dynamic modification, +80 Da), and cysteine (carboxamidomethylation, static modification, +57 Da) was allowed; precursor ions were required to be within *m/z* 0.6 of the peptide monoisotopic mass; fragment ions were required to fall within *m/z* 0.5 of the expected monoisotopic mass. IDPicker (Ma *et al.* 2009; Zhang *et al.* 2007) v2.5.180 was used to filter peptide matches with the following parameters: maximum FDR per result, 0.05; maximum ambiguous IDs per result, 2; minimum peptide length per result, 5; minimum distinct peptides per protein, 2; minimum additional peptides per protein group, 1; minimum number of spectra per protein, 2; indistinct modifications M 15.994 Da, C 57.05 Da; and distinct modifications S/T/Y 80 Da. IDPicker results were processed in Excel (Microsoft) to generate protein identification lists. Proteins identified as contaminants (*e.g.* keratin) were removed from the final protein list.

### Statistics of degree correlation

Wilcoxon rank-sum tests and Spearman correlation tests were performed using R to assess the correlation of the degree of a node in a specific network with any other feature used in this work.

### Genome-wide interaction predictions

Protein interactions among all of the fission yeast proteins were predicted. Each protein was assigned a GO superslim term. We used these terms in the following order to ensure that the most specific term was used: GO:0007005, mitochondrion organization and metabolism; GO:0007165, signal transduction; GO:0006350, transcription; GO:0016070, RNA metabolism; GO:000412, protein biosynthesis; GO:0006259, DNA metabolism; GO:0007049, cell cycle; GO:0006810, transport; GO:0044238, primary metabolism; and GO: 0008150, biological process.

## RESULTS

### Classification approach to predict protein interactions

We interpreted the protein prediction problem as a classification task on protein pairs that can be interacting or not. We required a training set to perform the classification that was composed of known interactions (positive training set) and a corresponding set of supposedly non-interacting pairs (negative training set), as well as a list of features. We constructed both a support vector machine (Cortes and Vapnik 1995) and a random forest (Breiman 2001) with the same input data and compared the results. These methods use an assembly of features from protein pairs to learn the difference between interacting and non-interacting pairs, and then score the new interaction predictions.

### Features used

As a basis for the machine learning approaches, we compiled two types of features, describing either the fission yeast proteins themselves (*e.g.* sequence-based features) or their interactions (*e.g.* co-expression). An overview of all features is presented in Table 1 and File S1. These features include physical position on the chromosome, physical properties of the protein (such as length, mass, and chemical properties), functional category information (through GO Superslim classification), as well as protein pair information, such as expression correlation over hundreds of different conditions, co-localization, distance along the chromosome, and the presence of genetic interactions (see *Materials and Methods* for more details).

**Table 1:** Description of features used in the prediction of the interactions

Feature Class	Features and Description of Data
Gene Ontology*^a^*	GO.0006259_DNA_METABOLISM
	GO.0006350_TRANSCRIPTION
	GO.0006412_PROTEIN_BIOSYNTHESIS
	GO.0006810_TRANSPORT
	GO.0007005_MITOCHONDRION_ORGANIZATION_AND_BIOGENESIS
	GO.0007049_CELL_CYCLE
	GO.0007165_SIGNAL_TRANSDUCTION
	GO.0008150_BIOLOGICAL_PROCESS
	GO.0016070_RNA_METABOLISM
	GO.0044238_PRIMARY_METABOLISM
Chromosomal position	Strand, chromosome, start and end positions
Distance from centromeres/telomeres	Absolute and relative distance
Gene physical properties	Length of the ORF, number of introns, length, and GC content of the first intron
Protein physical properties	Isoelectric point and mass of the protein (kDa), total and relative abundance of each amino acid in the protein, sulfur and nitrogen content, Codon Adaptation Index, protein length, codon bias, FOP frequency of optimal codons, and indexes of hydropathicity (Gravy score) and aromaticity (frequency of aromatic amino acids, such as phenylalanine, tyrosine, and tryptophan)
Protein localization	Protein localization in the cell and index of co-localization (Hayashi *et al.* 2009; Matsuyama *et al.* 2006)
Experimental gene properties	Experimental gene properties: mRNA half life, ribosome occupancy and density, mRNA levels, and Pol-II occupancy (Lackner *et al.* 2007)
Genetic interactions	Known genetic interactions from the BioGRID (Stark *et al.* 2011)
Pair physical features	Same strand, same chromosome, and distance on chromosome
Expression correlation	Pearson correlation of mRNA levels over about 100 different experimental conditions (Pancaldi *et al.* 2010)

More details can be found in File S1.

a We used the terms for a custom-built GO superslim classification.

### Model cross-validation

**Figure 1:**
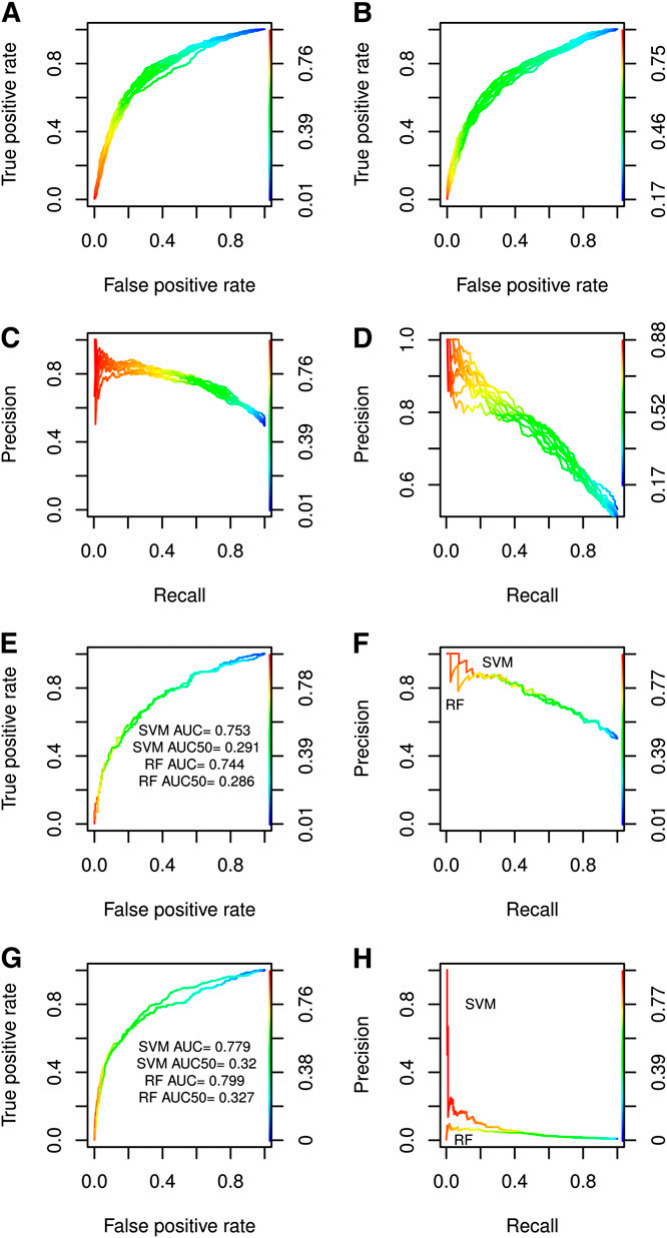
Model cross-validation and model testing on an independent test set. (A, B) ROC curves of 10 repeats with 2-fold cross-validation tests performed with SVM and RF, respectively. ROC curves show how the relationship between the true-and false-positive rate changes as a function of the probability threshold. If we are interested in making a few predictions with the smallest possible chance for errors, we should consider a high threshold. (C, D) Precision-recall curves corresponding to A and B. Precision-recall curves show the rate of correctly predicted interactions *vs.* the rate of predicted interactions. (E, F) Model testing using an independent fission yeast data set and an equally large degree balanced negative test set with the same proteins. (G, H) ROC and precision-recall curves for a second test set composed of the 204 high-confidence interactions and ∼32,000 random negative pairs, which are assumed not to be interacting. In this test, the ratio of positives to total pairs is similar to what we expect in predicting the whole interactome.

Both the SVM and RF classification methods output a probability for the interaction of any tested pair, which we interpreted as a score. Cross-validation is useful to assess the performance of the predictor on a subset of the data while the rest of it is used for training. Two-fold cross-validation, which uses half of the data for training and half for testing, was performed 10 times for both the SVM and the RF models. The average accuracy of the SVM was 0.76, with an AUC of 0.81 (compared with 0.5 expected by chance and 1 for perfect classification). The results for RF in this cross-validation test were marginally worse (accuracy, 0.73; AUC, 0.79; File S2). Considering predictions with a score above 0.5, the specificity (0.80 for SVM and 0.74 for RF) was better than the sensitivity (0.71 for both SVM and RF), suggesting that the false negatives were higher than the false positives at this threshold. We can see from the ROC curves (Figure 1, A and B) and precision-recall curves (Figure 1, C and D) that the results were robust with respect to different splitting of the data into training and test sets. We conclude that our machine learning approach manages to successfully predict many protein-protein interactions based on the features used.

### Model testing on proteins not present in training sets

Cross-validation results cannot estimate the power of the method to generalize to unseen data; hence, it is necessary to test it on a completely independent set of interactions. We report the classification performance on the test set with a one-to-one ratio between positive and negative pairs.

Figure 1 shows the ROC curve for SVM compared with RF (Figure 1E) with the corresponding precision-recall curve (Figure 1F). File S2 summarizes the statistics of the tests performed. RF and SVM achieved comparable results. In these tests, SVM showed higher accuracy (0.71) and higher AUC50 (0.291) compared with RF (0.68 and 0.286, respectively). The specificities ranged from 0.66 to 0.81, and the sensitivities ranged from 0.63 to 0.73. Generally, RF tended to produce fewer false positives, with the consequence of missing more of the true interactions compared with SVM.

### Test with imbalance between positive and negative pairs

Protein interaction networks are generally sparse; therefore, we expect a small fraction of real interactions among all possible protein pairs in fission yeast. To account for the excess of negative interactions, we also tested the performance of our method using a negative test set much larger than the positive test set. The results showed that, even with a large imbalance between the number of true and false interactions in the test sets, our methods can successfully limit the false-positive rate while correctly identifying the true positives. Although both the RF and SVM methods showed comparable performance (Figure 1, G and H), RF achieved a better accuracy (0.81) compared with SVM (0.75) and slightly better AUC50 (0.327 for RF and 0.32 for SVM).

### Feature importance

**Figure 2:**
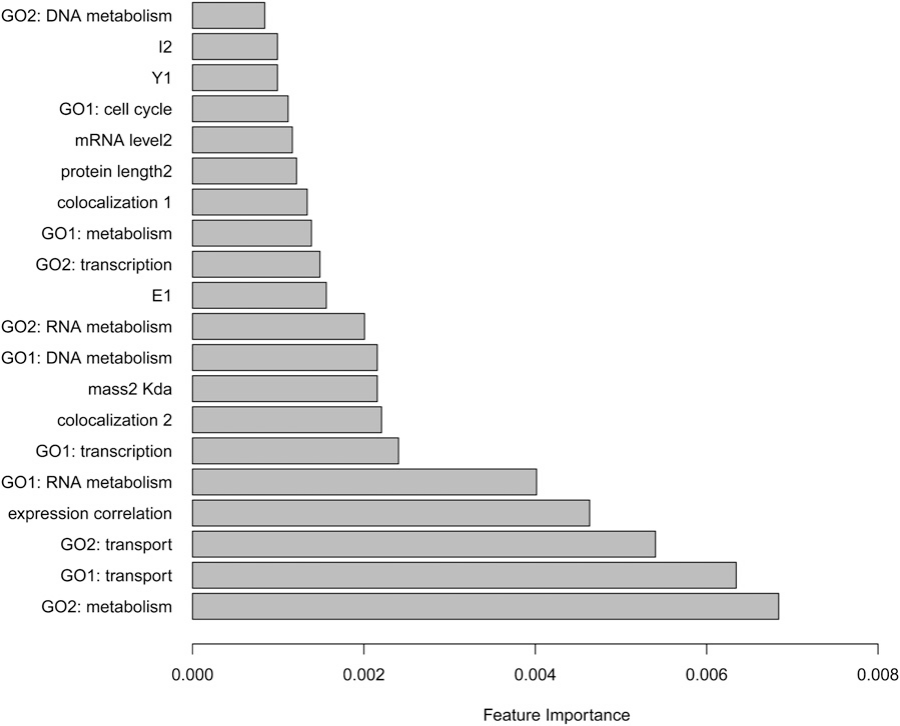
Importance of features in the RF classifier. Only the most important features are shown. Expression correlation and GO functional categories are the most important features, followed by protein length, mRNA levels, and protein co-localization (File S3; see File S1 for explanation of feature names). These values are the average of the importance of each feature in 10 realizations of the 2-fold cross-validation test.

We estimated the importance of each feature using a method that was proven to correctly take into account redundancy between the features and a mixture of feature types (Strobl *et al.* 2008). The features that are most influential in making the predictions are expression correlation; various GO terms, mostly related to metabolism and transport; and the length, abundance, and localization of proteins (Figure 2). Similar results were obtained by measuring the mean decrease in accuracy as the trees were being built (File S3). To quantify the predictive power of each feature alone, we conducted *t*-tests and Fisher’s tests estimating the significance of the differences between feature values in the positive and negative training sets. This analysis generally confirms our initial results (File S3).

### Predictions of SAGA complex interactions

Next we focused on the prediction of interactors of the SAGA complex. At a score threshold of 0.5, we only failed to predict two interactions with either SVM or RF: one between Ubp8 and Sus1 (Cuenca-Bono *et al.* 2010), and one between Ubp8 and Tra1. The consistency of these negative results between SVM and RF raises the possibility that these SAGA subunits are part of two different sub-complexes or modules with specialized functions. This possibility is in line with findings in budding yeast, which place Ubp8 and Sgf11 in a discrete functional module within SAGA (Ingvarsdottir *et al.* 2005). More recently, an analysis of deletion mutants for a few components of the fission yeast SAGA complex has also highlighted the presence of different modules within the complex. Intriguingly, we recover these modules to some extent when looking at the similarity of interaction profiles of the subunits. For example, we see the separate role of Ubp8 and Tra1, and the close link of Spt7 and Hfi1 and Sgf73 and Sgf29 (Helmlinger *et al.* 2011) (Figure S1). At a score threshold of 0.9, SVM predicted 92 interactions, 86 of which were part of the 171 total known SAGA interactions, whereas no interactions were predicted with RF.

RF scores generally tended to be lower than SVM scores so that choosing a specific score threshold led to a different number of predictions in the two methods. However, a correlation between the ranking of interactions according to SVM and RF was evident (Spearman r = 0.88, *P* < 10^−16^). To further check the specificity of our predictions, we calculated precision and recall curves for identifying the SAGA interactions from the total of the possible interactions of SAGA units with other proteins (Figure S1).

We predicted 132 and 126 additional interactions with SVM and RF, respectively, for which at least one of the proteins was in the control set. Notably, 105 of these interactions involving non-SAGA proteins were predicted both by SVM and RF, 28 of them with SVM scores > 0.8. The control proteins predicted to interact with SAGA components were characterized by involvement in chromatin and DNA damage repair (File S4). For example, many of the highest scoring interactions were between known SAGA proteins and Phf1, a histone H3-K9 demethylase. We therefore hypothesize that some of these predicted interactions could in fact be true and thus point to potential new SAGA components or to proteins transiently interacting with the SAGA complex. For example, we have found some evidence for Phf1, Cdc17, and Pku80 to be interacting with SAGA in other organisms (File S9). In conclusion, our classification approaches succeeded in recapitulating most of the SAGA complex, and they also suggested potential additional proteins that plausibly could associate with SAGA.

### Prediction of Cbf11 interactors

Cbf11 is a fission yeast member of the CBF1/RBP-Jκ/Suppressor of Hairless/Lag-1 (CSL) family of transcription factors. In metazoans, the CSL proteins serve as effectors of the Notch signaling pathway, which is critical for development. This protein family has been lost in budding yeast, and it seems to exert pleiotropic functions in fission yeast (Převorovský *et al.* 2009).

**Figure 3:**
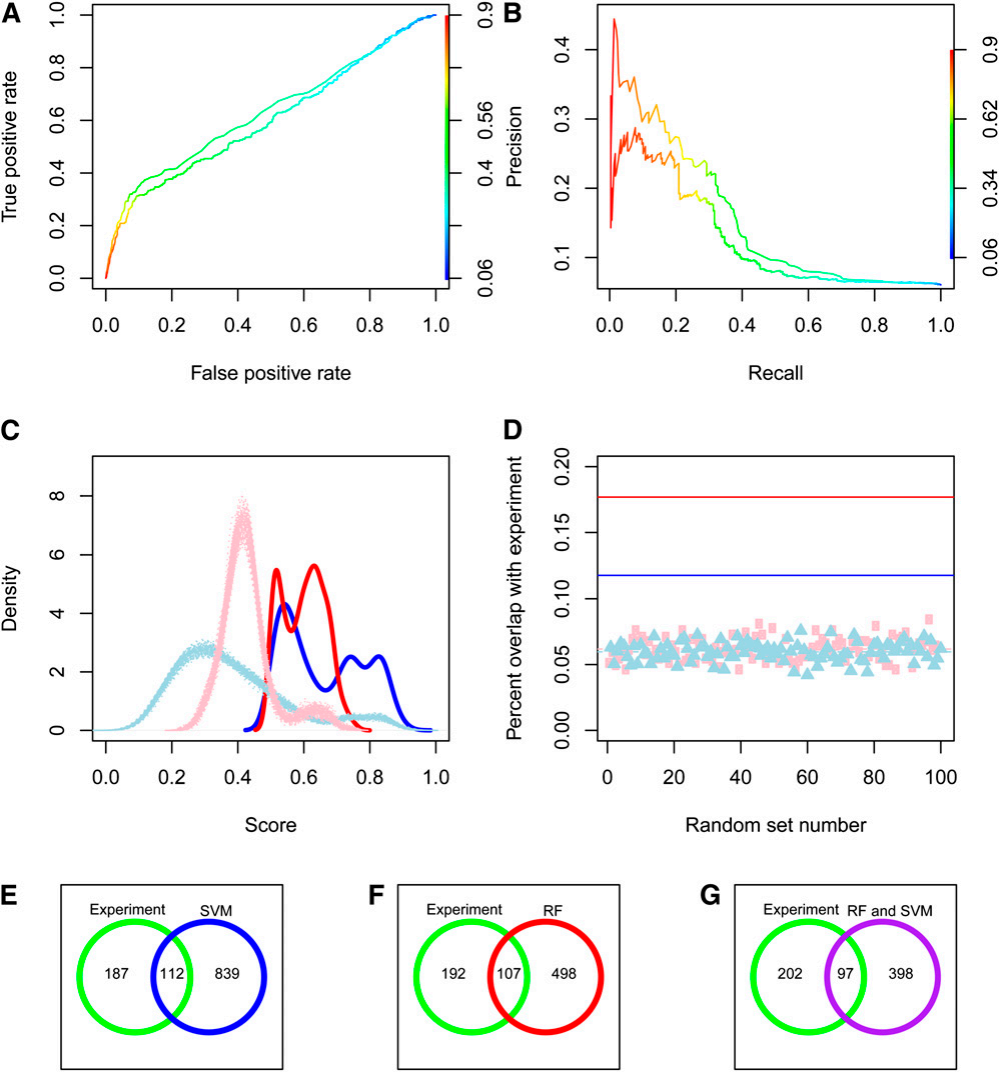
Estimation of performance of the predictions for the interaction partners of Cbf11. (A) ROC curves for RF (upper curve) and SVM (lower curve) obtained by comparing the predicted Cbf11 interactions with the experimentally verified targets. (B) Corresponding precision recall curves. (C) Distributions of the interactors predicted by RF (red line) and SVM (blue line) and of the corresponding 100 sets of genes picked at random from the fission yeast proteome (RF, pink line; SVM, light-blue line). (D) Overlap between the predicted interactors and the ∼300 experimentally verified targets (solid lines, RF, red; SVM, blue) and overlaps for each of the 100 random sets (RF, pink squares; SVM light-blue triangles). Lower panel: Venn diagrams showing overlaps between experimentally identified targets and the predictions, SVM (E), RF (F), and overlap of RF and SVM (G).

A purification experiment identified ∼300 potential components of the Cbf11 complex. The performance of the prediction compared with experimental results is shown in Figure 3 with ROC curves, precision-recall curves, and Venn diagrams for SVM and RF. It is possible that the experiment would itself have false negatives, as the experiments carried out in a specific experimental condition do not necessarily identify all the partners of a protein, especially the less stable ones. Notably, a significant correlation was found between the SVM prediction score and the number of peptides identified in the affinity capture-MS experiment (Spearman r = 0.25, *P* = 0.007), suggesting that our confidence scores could also reflect the strength of the experimental evidence. The scores obtained with RF, however, did not correlate with the peptide count (*P* > 0.5).

SVM and RF contributed 15 and 10 unique predictions, respectively, that were confirmed experimentally. Thus, the two methods were somewhat complementary and, if used together, may provide better coverage of true predictions. On the other hand, using the overlap of predictions from both SVM and RF provides a more conservative and, hence, more reliable list of protein interactions that could be used as a starting point for further investigations.

The Cbf11 interactors predicted were significantly enriched for the experimentally determined targets, both in the case of SVM (Fisher’s exact test, *P* = 10^−14^), RF (Fisher’s *P* = 10^−28^), and in the overlap of the two methods (Fisher’s *P* = 10^−28^). So far, there are no known genetic interactions for Cbf11 and no functional interactions due to the protein not being conserved in budding yeast. For this reason, our method for predicting its physical association partners can only be compared with selecting proteins at random from the whole genome. The results of this test are shown in Figure 3, C and D, revealing that the scores of the predicted targets are much higher than scores for other equally large sets of genes picked at random (Figure 3C). On closer analysis, two peaks can be identified in the score distribution of the predicted set, suggesting that by considering only scores above 0.7, we are likely to eliminate many of the false positives. Our predictions offer almost three times as many verified targets as random lists of genes. The presence of a peak of high scores in the random sets’ distribution (continuous curves in Figure 3C) and the overlaps between random sets and the verified targets (circles in Figure 3D) are consistent evidence for the likely presence of a small number of real interactors or false positives in the random sets, which can be expected.

We performed a GO term enrichment analysis on the predicted Cbf11 interactors as well as on the experimentally identified interactors (File S4). Both these lists were significantly enriched for transcriptional control, RNA processing, and chromatin remodeling; these enrichments were particularly significant among the 97 proteins that were both predicted by the two techniques and experimentally confirmed as Cbf11 interactors. The terms that we found in the enrichment are of finer level compared with the ones used as features in the training, so this enrichment is not trivial.

The proteins that are not predicted to interact but are experimentally found to co-purify with Cbf11 were only weakly enriched for organelle and chromosome organization and for metabolic proteins, suggesting that some of these interactions could be false positives of the affinity capture-MS method or background interactions. Consistent with the latter hypothesis, more than 20% of these interactions were with proteins that could be considered non-specific background (*i.e.* identified in no TAP tag negative controls or in most other unrelated TAP/LC-MS/MS analyses performed in our laboratory; File S4). Notably, there were also 398 interactions predicted with both SVM and RF, but not confirmed experimentally, which showed strong enrichment for the same GO terms that characterize the experimentally verified interactors (File S4). These genes showed significantly lower mRNA levels (Wilcoxon rank-sum test, *P* = 10^−5^) and shorter mRNA half-lives (*P* = 10^−5^), raising the possibility that they escaped the purification steps or were below the detection sensitivity of the affinity capture-MS approach. We conclude that our list of predicted targets contains approximately one third of the experimentally verified Cbf11 interactors and is not biased by protein levels that could affect the affinity capture-MS data.

**Figure 4:**
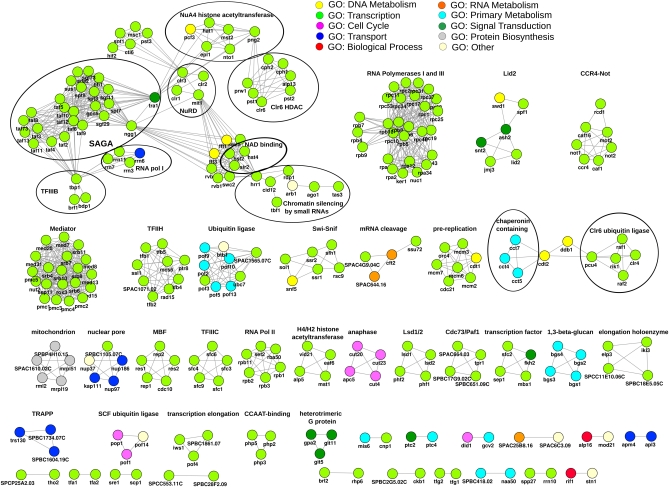
Known interactions among predicted Cbf11 interactors. The proteins shown are the subset of the predicted Cbf11 interactors that are annotated to be part of a complex in fission yeast. Many complexes are predicted to interact with Cbf11. In some cases, almost all the subunits are predicted interactors (File S4).

Moreover, our list of predictions contains whole complexes that could interact through some of their units with Cbf11. Figure 4 shows the predicted Cbf11 interactors that are annotated to be part of a complex in fission yeast. In some cases, like the SAGA complex, the vast majority of the subunits of the complex are predicted to interact. In others, like the pre-replication complex, only a subset is predicted. Looking at these complexes shows that, within the set of predicted interactions, clear biological modules can be identified. Using a relatively low cut-off of 0.5 (applied to SVM and RF) leads to as many as 951 predicted interactors, raising the possibility that these complexes are just predicted by chance. However, even raising the SVM threshold to 0.7 (as suggested by the score distributions of RF and SVM shown in Figure 3C), we observe many components of the same complexes among the predicted interactors. Moreover, these complexes were also present in the list of experimentally verified Cbf11 interactors (File S4). The general theme of chromatin remodeling among Cbf11 interactors, with an emphasis on histone modification and silencing, suggests a role for this transcription factor in epigenetic processes, which could explain the wide range of phenotypes that have been associated with it.

### Validation of other lower-confidence complex interactions from the literature

Using GO annotations, a curated list of fission yeast complexes can be compiled (GO complex interactions). Assuming that all members of a complex interact with each other and not with members of other complexes, we constructed a set of 28,771 interactions and 1,374,879 non-interactions. Using a threshold of 0.5 for both SVM and RF, we confirmed 14,720 of the interactions and 1,191,506 of the non-interactions, with a precision of 0.07 and an accuracy of ∼0.86 (Figure S2). Importantly, the assumption that there are no interactions between units of these different complexes and that all complex units interact directly is very likely wrong, showing that these estimates are only very rough approximations of the real performance.

### Validation of new high-confidence interactions

Looking at interactions from the GO complex list described above, which are also verified in a study for interaction of chromatin-associated proteins (Shevchenko *et al.* 2008), we predicted 39 out of 58. Additionally, as of April 2011, 15 more pairs can be added to the list of 204 fission yeast interactions, with two different lines of evidence (capture and Y2H), which was used for our testing in 2010. Of these, 11 are predicted by both RF and SVM, and 5 have an SVM score higher than 0.8 (File S5 and Figure S3).

### Node degree is correlated with protein length and not abundance

We then checked whether particular types of proteins were predicted to have many interactors. To answer this question, we examined the tendency of proteins with specific feature values to have many interacting partners in three different networks: (1) a network of protein interactions in fission yeast from databases (including the ones mapped from budding yeast); (2) the filtered subset of this known network used as our positive training set (two independent lines of evidence); and (3) the network of predicted interactions with scores higher than 0.9. In the protein interaction network built from databases, we find that proteins with more connections have a higher relative polymerase II occupancy (Spearman r = 0.075, *P* = 10^−5^), suggesting that they are relatively more abundant. However, this is not the case in either of the other networks. It is hard to determine whether this trend is biologically relevant. The correlation of the degree with the protein level is to be expected in experiments, where capture techniques are more sensitive to abundant proteins (especially with high-throughput methods such as mass spectrometry), but it was not found in our predictions or in a high-quality dataset. This finding is, therefore, consistent with a bias in the experimental data. Interestingly, highly connected proteins tended to be longer, both in the known network (Spearman r = 0.09, *P* = 10^−7^) and in the predicted network (Spearman r = 0.16, *P* = 10^−20^) (File S6), which could reflect the presence of more interaction domains on longer proteins.

### Global prediction of the protein interaction network in fission yeast

**Figure 5:**
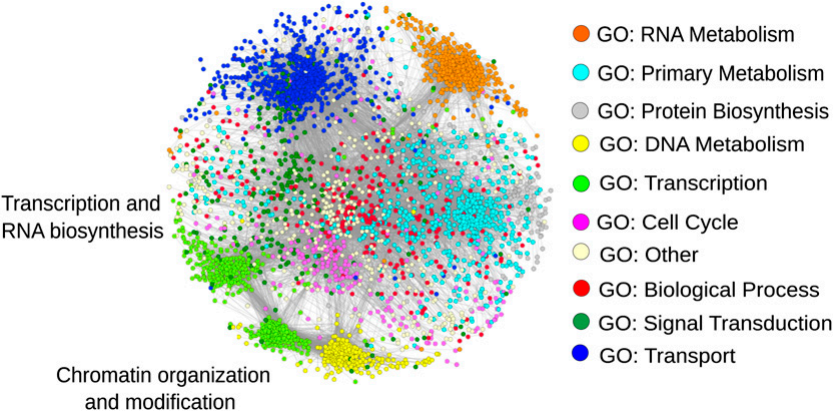
Predicted network with interactions confirmed by RF and SVM. The network includes 3438 proteins and 37,325 interactions for clarity. The Cytoscape organic layout was used for visualization (Smoot *et al.* 2011). The emerging clusters reflect highly connected portions of the network. These clusters coincide with the GO superslim functional categories of the proteins and are color-coded as indicated (Ashburner *et al.* 2000).

Having assessed the accuracy of our prediction method, we calculated probability scores for all possible fission yeast protein interactions. The almost 5000 proteins can potentially interact in over 12.46 million possible pairs. We performed the predictions with both SVM and RF and used the overlap as a conservative estimate of the fission yeast protein interactome. We chose a score threshold (0.87) that resulted in a number of interactions comparable to the ∼60,000 interactions known for budding yeast (Stark *et al.* 2011) and within other estimates (Hart *et al.* 2006). As we have seen while examining the predictions for Cbf11 interactions against experimental data, the score assigned by the SVM to each pair appears to be a reliable measure of the strength of the interaction and an indication of whether it is direct. The network of all interactions passing our filtering criteria showed coherent modules characterized by GO terms (Figure 5). This finding may reflect that GO terms are features in the classification, but we still observed these modules to some extent when the GO terms were omitted from the training (Figure S4). This result indicated the presence of redundant information in the different features used.

Although budding yeast interactions were used in the training sets, the predictions were based entirely on fission yeast features. The resulting predicted interactome, therefore, offers a species-specific picture. The light green GO:transcription cluster appears to be split into two sub-clusters (Figure 5). A GO term enrichment analysis confirms the difference between the two sets of proteins: the smaller one enriched for chromatin organization and modification, and the larger one enriched for transcription and RNA biosynthesis (data not shown). This example shows how the clustering of the predicted interactome is not uniquely determined by the GO features used in the training, confirming the importance of the other features. It also indicates that our approach provides insight into the higher-order organization of biological processes within the cell that might be incompletely described by GO.

The interaction score provided by the SVM can be exploited using two complementary approaches. First, we can use a large number of predicted interactions to explore the context of a biological pathway, aware of the presence of false positives. Alternatively, we can select a few high-confidence predictions and verify them experimentally. It should be mentioned that most of our false positives are likely to be functionally related proteins. Filtering the positive predictions for a protein of interest based on the presence of compatible interaction domains on the proteins would help to distinguish more confidently the true direct physical interactions. In the following, we used the predicted interactome to investigate the stress response sub-network.

### Stress response sub-network

**Figure 6:**
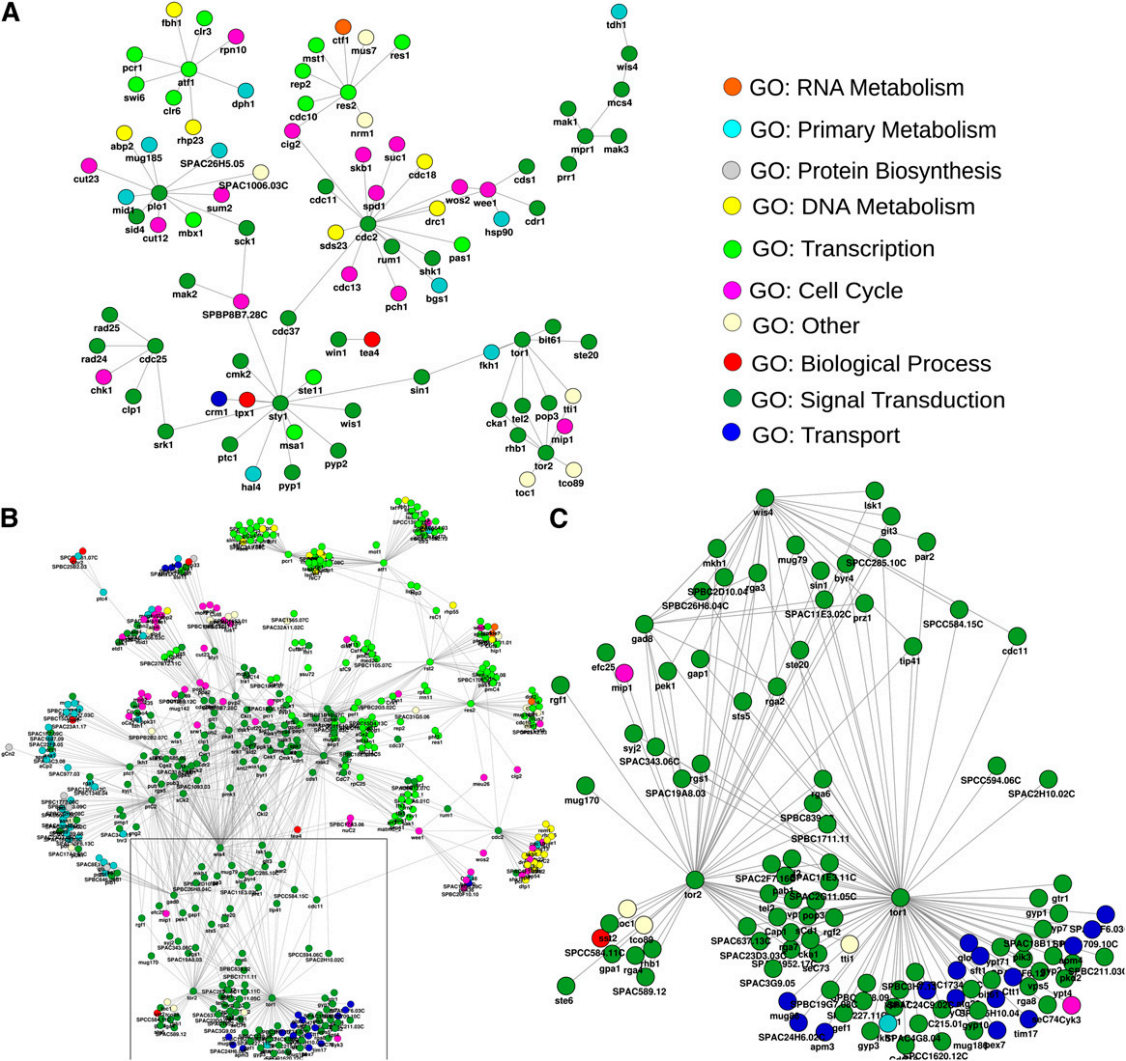
The stress response sub-network. (A) Known interactions of stress-related genes (from BioGRID). (B) Network in A expanded using our predictions. (C) Detail of the network for Tor1 and Tor2 kinases.

The cellular stress response is a universal mechanism of pivotal significance for both cellular physiology and pathology. It represents a defense reaction of cells to maintain homeostasis and survive environmental challenges. The response of fission yeast to environmental stress has been the subject of multiple studies that provide a framework to explore the predicted protein interaction network (Figure 6). We therefore focused on the neighborhood of proteins related to stress response. Any change of the environment activates a core environmental stress response (CESR) (Chen *et al.* 2003, 2008; Lopez-Maury *et al.* 2008). The large gene expression response is bidirectional, inducing cellular stress defense mechanisms as well as repressing growth-related processes.

We compiled a list of 32 proteins known or suspected to be involved in stress response from the literature. Most of these proteins are highly conserved, with some of them having multiple paralogs in budding and fission yeast. Figure 6 shows the sub-network of fission yeast proteins that are recorded in databases (Figure 6A) or were predicted here (Figure 6B) to interact with any of the selected 32 stress-response proteins, defining a stress response network. Besides several previously reported interactions [*e.g.* the one between Prr1 and Mpr1 (Aoyama *et al.* 2001; Nakamichi *et al.* 2003)], our network contains numerous potentially new interactions (*e.g.* between Prr1 and Pka1, Cyr1 and Ssu72). As examples for how our predictions can be used for the generation of new biological hypotheses, we highlight below a few of the predicted interactions and discuss them within their biological context.

### Predicted TOR interactions

The evolutionary conserved target of rapamycin (Tor) pathway mediates fundamental cellular activities, such as translation, spatial and temporal growth, and cell-cycle progression in response to nutrient signals. Fission yeast Tor1 and Tor2 proteins are serine/threonine kinases of the phosphatidylinositol kinase–related family (Lopez-Maury *et al.* 2008) that function in the multiprotein complexes TORC1 and TORC2 (Bjornsti and Houghton 2004; Petersen 2009). Although the Tor signaling pathway is of great interest and subject to intense research in several model organisms, the list of known molecular players is far from complete. Proteins that were predicted to interact with both *S. pombe* Tor kinases are involved in cell shape determination; cortical patch localization and cell polarity; septum formation; vacuole targeting and sorting; endocytosis; and autophagy (Figure 6C and Table 2). A large number of predictions included GTPases and GTPase activators or inhibitors, such as the ras activator GAP protein Byr4 (Song *et al.* 1996). GTPases (*e.g.* of the Rho family, present in our list) are central for the regulation of spatial cytoarchitecture (polarity and growth) via regulation of cytoskeletal elements (Perez and Rincón 2010), autophagy (Mukaiyama *et al.* 2010), and trafficking (Burguete *et al.* 2008). Our data raise the possibility that these GTPases may be regulated by Tor. Interestingly, the predictions also included proteins, such as Rab GTPases, that may themselves regulate Tor signaling (Tatebe *et al.* 2010). Although autophagy mechanisms have been described for *S. pombe*, our understanding of this process and its regulation has remained elusive. For example, Tor kinases are localized in distinct membrane-associated protein complexes (Wedaman *et al.* 2003), but the nature of these complexes is unknown. Using our method, Tor1 kinase was predicted to interact with Atg20 and Atg24 (sorting nexins), providing clues for the composition of such complexes. In addition, our list includes one of the two pH sensor bro1 domain proteins involved in ascospore formation and protein processing (Table 2 and File S7). These predictions thus provide an intriguing list of candidates to follow up with targeted wet-lab experiments. Given the strong conservation of the Tor pathway, our predicted interactions should also be fruitful to pursue in other organisms.

**Table 2:** Predicted interactions for both Tor1 and Tor2 obtained with both RF and SVM (score > 0.87)

Description (GeneDB Annotation)	Common Name	Systematic ID
Adenylyl cyclase–associated protein Cap1	Cap1	SPCC306.09c
Arrestin Aly1 related	SPBC839.02	SPBC839.02
Autophagy-associated protein	SPBC1711.11	SPBC1711.11
Cytoskeletal signaling protein	SPAC637.^13^C	SPAC637.^13^C
GTPase activating protein	SPAC1952.17c	SPAC1952.17c
GTPase activating protein	SPAC23D3.03c	SPAC23D3.03c
GTPase activating protein	SPAC3G9.05	SPAC3G9.05
Guanyl-nucleotide exchange factor (predict.)	SPAC11E3.^11^C	SPAC11E3.^11^C
Guanyl-nucleotide exchange factor Sec73	Sec73	SPAC19A8.01c
Meiotically upregulated gene Mug79	Mug79	SPAC6G9.04
Regulator of G-protein signaling Rgs1	Rgs1	SPAC22F3.12c
RhoGEF Rgf2	Rgf2	SPAC1006.06
RhoGEF Scd1	Scd1	SPAC16E8.09
Rho-type GTPase activating protein Rga2	Rga2	SPAC26A3.09c
Rho-type GTPase activating protein Rga3	Rga3	SPAC29A4.11
Rho-type GTPase activating protein Rga6	Rga6	SPBC354.13
Rho-type GTPase activating protein Rga7	Rga7	SPBC23G7.08c
RNB-like protein	Sts5	SPCC16C4.09
Scaffold protein Scd2	Scd2	SPAC22H10.07
Sorting nexin Mvp1	Mvp1	SPAC3A11.06
SPRY domain protein	SPCC285.10c	SPCC285.10c
Type 2a phosphatase regulator Tip41	Tip41	SPCC4B3.16
Two-component GAP Byr4	Byr4	SPAC222.10c

### Connections with disease

The mTOR pathway in humans is an important contributor to disease in various cancers, including hematologic malignancies and neurodegenerative diseases, such as Huntington disease (Wang *et al.* 2009b), via still unclear mechanisms of suppression of autophagy and myopathies (Risson *et al.* 2009). Tor signaling is shown to be directly implicated in neurodegenerative disorders (Wang *et al.* 2009b) and may include additional interactions and pathways. Constitutive activation of mTOR is shown to sensitize cells to genomic damage (Lee *et al.* 2007). A protein-protein interaction map for Tor is therefore of significance for understanding the action mechanisms of this kinase in pathological conditions. Human homologs of proteins predicted with our tool to physically interact with *S. pombe* Tor1 kinase are implicated in disease and have Online Mendelian Inheritance in Man (OMIM) entries related to various cancers, neuronal diseases or syndromes, myopathies, and porphyria (Table 3).

**Table 3:** Predicted interaction partners of the Tor kinases whose orthologs are associated with human disease

Human Protein	*S. pombe* Protein	Disease	Reference	Comment
CLN3	Btn1	Batten disease (MIM ID #204200)	Rakheja *et al.* (2008)	Genetically interacts with the core stress signaling pathways; *Drosophila* lacking *cln3* function are hypersensitive to oxidative stress, whereas overexpression of *cln3* protects from such stress (Tuxworth *et al.* 2011). Disruption has been linked to mTOR expression (Cao *et al.* 2006).
PDHB	Pdb1	Pyruvate decarboxylase deficiency (MIM ID #312170)		Related to cerebral ataxia.
CYCS	Cyc1	Huntington disease (MIM ID #143100) and thrombocytopenia 1 (MIM ID #313900)	Wang *et al.* (2009b)	Cytochrome C, a protein participating in electron transport chain in mitochondria; also localized in the cytoplasm.
PDK3	Pkp1	Congenital myopathy (MIM ID #300580)		Mitochondrial pyruvate dehydrogenase (lipoamide) kinase.
PPOX	Hem14	Porphyria (MIM IDs #176200, #176000)		Penultimate enzyme of haem biosynthesis targeted to mitochondria.

The predicted Tor interactions with proteins that function in mitochondria might be unexpected. Although a possible relation seems unlikely, it is already reported that Tor signaling is related to mitochondrial function, and mutations that cripple Tor signaling have effects on mitochondrial respiration. Indeed, increased oxygen consumption is a signature of Tor mutants (Bonawitz *et al.* 2007; Pan and Shadel 2009). Our protein-protein interaction predictions suggest relations and mechanisms that could be used for further disease studies and potential targeted drug design.

### Predicted Mak1/2/3 interactions

Histidine kinases mediate a histidyl-aspartyl phosphorelay, also known as a two-component system (TCS). We predicted the *S. pombe* histidine kinases Mak1, Mak2, and Mak3 to be central in the stress response sub-network (File S7). Histidine kinases are reported to have important roles in bacteria, other fungal species, and plants in regulation of osmotic and oxidative stress response (Desikan *et al.* 2008; Krell *et al.* 2010; Motoyama *et al.* 2005; Santos and Shiozaki 2001; Urao *et al.* 2001), functioning as parts of TCSs. While mammals developed complex signaling systems involving serine/threonine/tyrosine kinases for stress response, bacterial and fungal TCSs represent a simple but elegant prototype of signal transduction machineries. Due to their significance in bacterial and fungal virulence and the development of antibiotic resistance, they are of great interest in the development of antimicrobial and antifungal agents (Rowland and King 2007; Schreiber *et al.* 2009; Stephenson and Hoch 2002, 2004). Fission yeast Mak2/3 have been reported to play important roles as oxidative stress sensors by mediating phosphorylation of the Atf1 transcription factor via the Sty1 kinase (Buck *et al.* 2001). However, the role of Mak1 is different and required for Pap1- and Prr1-dependent transcription (Buck *et al.* 2001). The only histidine kinase in budding yeast, Sln1, plays a role as an osmosensor in response to osmotic stress (Posas *et al.* 1996). We predicted the three *S. pombe* histidine kinases to interact with 127 proteins that were enriched for signal transduction, cytokinesis, septation, RNA metabolism, and response to chemical stimuli (File S7). Within these 127 proteins, we found 41 genetic interactions and 37 physical interactions inferred from budding yeast (as documented in BioGRID). As many as 18 transcription factors were predicted to be interacting with the Mak proteins, 6 of them specifically with Mak2 (Atf31, Hsr1, Rsv1, Res1, Moc3, and Prr1). We conclude that the predicted stress response sub-network is a useful framework to generate specific hypotheses for this important biological process.

### Predicted argonaute interactions

Cellular response to stress is highly dynamic, allowing the cells to effectively counteract diverse stresses. It is evident from mammalian studies that siRNAs are a new class of genes involved in stress response, apart from regulation of gene expression and anti-virulence action (Babar *et al.* 2008). siRNAs are of immense interest as a gene function research tool but also as active agents in a new variety of therapeutics (Walton *et al.* 2010). Interestingly, *S. pombe* has the machinery for siRNA production and processing that *S. cerevisiae* lacks and has, thus, emerged as a useful model for RNA interference: it has single-copy genes for components of the RNAi pathway, such as argonaute, dicer, and RNA-dependent RNA polymerase (RdRP) (Bühler *et al.* 2008). Functions for RNAi revealed in *S. pombe*, such as heterochromatic silencing and chromosome segregation, are likely to be ancient because they are shared with other eukaryotes (Martienssen *et al.* 2005). Importantly, a link between RNAi and heterochromatin integrity is reported (Bayne *et al.* 2010). It is therefore interesting to examine physical interactors of proteins actively involved in this process, to gain insight on different mechanisms of gene regulation and stress response. argonaute 1 (Ago1 or eIF2c) is required for siRNA-mediated gene silencing in a range of biological models. However, only a few interactors of Ago1 are known. Using our tool, we identify a series of proteins involved in chromatin regulation as Ago1 interactors, notably numerous SAGA complex components, the histone deacetylases Hst2 and Sir2, and the chromatin silencing protein Clr2. Sir2 is involved in silencing at centromeres and telomeres (Freeman-Cook *et al.* 2005) and in processes that affect replicative lifespan (Wang *et al.* 2009b). Interestingly, Sir2 in *S. cerevisiae* is associated with the polarisome and plays an important role in segregation of damaged proteins between mother and daughter cells and the modulation of replicative lifespan (Liu *et al.* 2010), and it may have beneficial roles against neurodegeneration (Gan and Tang 2010). It would be interesting to elucidate the potential link between Sir2 actions and the siRNA machinery.

## DISCUSSION

We present the first approach to predict physical protein interactions in fission yeast based on a large number of experimental and sequence features. The expression correlation, functional characterization, and length of the interacting proteins emerge as the major factors for the predictions. The method can produce predictions even for proteins that lack functional annotation, as was demonstrated for Cbf11. Although we trained our method to identify physical interactions, the lowest-scoring predicted partners are likely to be proteins that are at least functionally interacting with the protein of interest. We used a training set that included the overlap between two very different types of evidence of interaction to reduce the number of false positives. In theory, proteins that are shown to interact both by Y2H and other capture methods should be stable and direct interactors, but we expect to find positive predictions even for unstable and co-complex interactions. Including a very coarse version the Gene Ontology in our features suggests that some of our false positives correspond to functional interactions. In fission yeast, even functional interactions are poorly characterized, especially the ones involving non-conserved proteins, such as Cbf11. Our method can hence also be used, to some extent, to explore the functional network neighborhood of uncharacterized proteins. Different training sets would need to be employed to improve the performance of this task, which is beyond the purpose of this work. A flexible use of the prediction scores should help in changing the focus from an exploratory list of possible interactions to a more conservative list. For an even stricter filter on proteins that interact physically and directly with the protein of interest, an assessment of the presence of compatible domains could be carried out on the predicted list.

### The usefulness of RF and SVM predictions

Having used SVM and RF in all analyses allows us to compare the two techniques. There is an advantage in using both techniques as each produced unique predictions that were verified experimentally. The overlap of predictions made by SVM and RF can be used as a high-confidence set. Generally, RF produces fewer predictions than SVM, eliminating both false positives and true positives and leading to better ROC and precision-recall curves. However, according to our validation by affinity capture-MS experiments, the probabilities returned by the SVM algorithm seem to correlate weakly but significantly with the peptide counts, a useful proxy for the strength of the experimental interaction. In the case of RF, this correlation was not significant.

### Choice of threshold for interaction score

An important issue in establishing a correct use of these predictions is to understand how changing the threshold affects their accuracy. To this end, we calculated our performance measures on the imbalanced sets for two additional thresholds: 0.7 and 0.9 (File S2). For example, the accuracy for the RF method is 0.81, 0.71, and 0.75 for thresholds of 0.5, 0.7, and 0.9, respectively, whereas the SVM method records accuracies of 0.77, 0.92, and 0.97 respectively. The large difference between SVM accuracies at 0.5 and 0.7 thresholds suggests that 0.5 is probably too low a cut-off. For the RF case, on the other hand, a threshold of 0.5 effectively limits the number of false positives, as shown by a high accuracy. Notably, the score assigned by SVM to Cbf11 interactors correlates significantly with the strength of the experimental evidence for these interactions. The classification underlying the SVM provides a score which should reflect the confidence of the predictions, whereas RF results do not guarantee this. We suggest that users of our tool consider the ranking of the predictions and determine an appropriate threshold based on the purpose of the prediction. Using the data provided in File S2 should further guide the interpretation of the predictions.

### Performance in recapitulating the SAGA complex and characterizing Cbf11

Almost all interactions within the SAGA complex are recapitulated, and additional plausible connections with other proteins are predicted. Of course, in the case of this well-annotated complex, most of these results could be obtained by mapping the budding yeast SAGA complex members into fission yeast orthologs. However, having eliminated all SAGA proteins from the training, we demonstrated how the method can be used to reconstruct uncharacterized complexes.

Moreover, we predicted one out of three interactions for Cbf11 that were produced as hits from an experimental approach involving affinity (TAP) purification followed by mass spectroscopy. In this way, a role in chromatin-related processes is suggested for this uncharacterized transcription factor. Many of our predicted targets that are not found in the experimental hit lists were enriched for similar specific GO processes and are likely to be at least functionally related to Cbf11. Moreover, we showed how some low-abundance proteins could be false negatives of the experiment and stressed the fact that high-abundance proteins can bias specific experimental approaches.

### General considerations on method performance

There are no other predicted physical interaction networks for fission yeast, with the only available functional interactions available from STRING (Szklarczyk *et al.* 2011) likely to miss important species-specific subsets of the network. A comparison of our predictions with the current version of STRING for fission yeast shows a reassuring correlation of the STRING combined scores with our predicted scores for SVM (Spearman r > 0.25) and for RF (r > 0.2). We predict 67% of the pairs with a STRING score > 800/1000 but only 46% of the pairs with a STRING score > 200/1000, showing that our method is mostly capturing physical interactions (File S8).

Using interaction domain information as well as sequence features, Yu *et al.* (2010) predicted budding yeast interactions reporting AUCs of up to 0.77, whereas we obtained AUCs of around 0.8 without including any domain information. This could be due to our careful construction of the training sets, which included only interactions with at least two kinds of experimental evidence for physical interaction.

Of the 15 new interactions that were added with two lines of evidence to BioGRID since the start of this project, 11 had been predicted by both our methods.

Estimating the real size of the interactome is not trivial and by necessity renders the accurate estimation of our method’s performance subject to large error margins (File S10). The low precision recorded (2%, reaching 7% when taking only the top 10% of predictions) can be attributed to this lack of knowledge and is characteristic of predicted networks, which can still be of use to guide hypothesis generation and experiments. The experimental validations presented in this article offer a practical example of the validity of the method and show possible uses of our predictions.

### New interactions suggested by the predicted fission yeast interactome

To demonstrate the biological use of the obtained interactome, we discussed predicted partners of the Tor proteins, which are involved in the regulation of growth and stress response, of the histidine kinases Mak1/2/3, which are involved in sensing of environmental perturbations, and of the only argonaute protein present in fission yeast. Although such predictions for physical interactions contain inevitable false positives and false negatives, we conclude that the predicted protein network can provide a useful framework to explore biological processes, expand the functional network around a protein of interest, and develop hypotheses to guide wet-lab experiments.

### A new resource for the fission yeast community and beyond

We believe that this tool will be of particular use to the yeast community to generate hypotheses and to investigate the role of yet uncharacterized proteins in fission yeast, like Cbf11, for which no physical interaction information is available and for which even functional interactions are unknown (Szklarczyk *et al.* 2011). We make our predictions available online for perusal by colleagues through a user friendly network visualization tool (http://www.bahlerlab.info/PInt/).

## Supplementary Material

Supporting Information
