# Supplementary material for: Predicting the Fission Yeast Protein Interaction Network
Source: G3 (Bethesda). 2012 Apr 1;2(4):453–67. doi: 10.1534/g3.111.001560 (PMC3337474; doi:10.1534/g3.111.001560)
Supplement: Supporting Information [file supp_2_4_453__index.html]

Supporting Information 

# Predicting the Fission Yeast Protein Interaction Network

## Supporting Information for Pancaldi *et al.*, 2012

**Files in this Data Supplement:**

- Supporting Information - Figures S1-S4 and Files S1-S10 (PDF, 1.4 MB)
- Figure S1 - Analysis of predictions for fission yeast SAGA complex units (PDF, 133 KB)
- Figure S2 - Comparison of SVM, RF and average SVM-RF predictions with the curated list of complexes in fission yeast (main text) (PDF, 98 KB)
- Figure S3 - Screenshot for predictions of all interactions of proteins which have new reported high-confidence interactions since 2010 (PDF, 673 KB)
- Figure S4 - Interactome obtained by eliminating Gene Ontology features for the training (PDF, 444 KB)
- File S9 - Supporting text (PDF, 51 KB)
- File S10 - Supporting text (PDF, 96 KB)
- File S1 - Supporting data (.xls, 38 KB)
- File S2 - Supporting data (.xls, 38 KB)
- File S3 - Supporting data (.xls, 92 KB)
- File S4 - Supporting data (.xls, 1.3 MB)
- File S5 - Supporting data (.xls, 19 KB)
- File S6 - Supporting data (.xls, 52 KB)
- File S7 - Supporting data (.xls, 528 KB)
- File S8 - Supporting data (.xls, 105 KB)
