# Supplementary material for: Predicting the Fission Yeast Protein Interaction Network
Source: G3 (Bethesda). 2012 Apr 1;2(4):453–67. doi: 10.1534/g3.111.001560 (PMC3337474; doi:10.1534/g3.111.001560)
Supplement: Supporting Information [file supp_2.4.453_001560SI.pdf]

Supporting Text and Figures for:

Predicting the fission yeast protein interaction network

Vera Pancaldi[1], Ömer S. Saraç[2], Charalampos Rallis[1], Janel R. McLean[3], Martin Převorovský[1], Kathleen Gould[3], Andreas Beyer[2], Jürg Bähler[1] 2

[1] Department of Genetics, Evolution & Environment and UCL Cancer Institute, University College London, London, Darwin Building, Gower Street, London WC1E 6BT, UK

[2]Cellular Networks and Systems Biology, Biotechnology Center - TU Dresden, Dresden, Germany

[3]Howard Hughes Medical Institute and Department of Cell and Developmental Biology, Vanderbilt University School of Medicine, Nashville, TN 37232, USA

Files S1- S8

Supporting Data

Files S1-S8 are available for download at

http://www.g3journal.org/lookup/suppl/doi:10.1534/g3.111.001560/-/DC1

File S9

Phf1 is known to interact with Ezh2, orthologue to the fly Polycomb group protein Enhancer of zeste (O'CONNELL *et al.* 2001). In fly, Pcl (Polycomblike) is an orthologue of Phf1 known to interact with Enhancer of zeste, which itself interacts with orthologues of members of the SAGA complex (Pcaf, Ada2b, …). This makes a link between Phf1 and the fission yeast SAGA complex very plausible. In human, Phf1 is known to interact with Ku80 (related to pku80, which we also predict to associate with the SAGA complex) (HONG *et al.* 2008). Moreover, the fly homologue of Cdc17 (CG5602) is also predicted to indirectly interact with polycomb group proteins and a few of the SAGA orthologues in STRING (SZKLARCZYK *et al.* 2011). Information collected with the use of iHOP (http://www.ihop-net.org/).

File S10

Our incomplete knowledge of the fission yeast interactome makes it impossible to know how many real interactions are within our negative sets. If we consider the expected size of the fission yeast interactome we can estimate how many true interactions we would expect in a specific number of random draws. For every 12.5 million pairs we expect around 60000 to be true, a ratio of 0.006. Hence, if we assume the network is equally dense in different regions, we expect 0.006 real interactions for every pair that we draw. Since we have ~32000 supposedly negative interactions in our negative test set, drawn at random, up to around 200 could be potentially true. If that were the case, our estimates of True Positives and False Positives would change drastically (given that the known true positives are only 204 adding 200 doubles this number) doubling the precision from 2% to 5%.This highlights how big the error margin on these estimates is in the absence of a completely known interactome.

HONG, Z., J. JIANG, L. LAN, S. NAKAJIMA, S. KANNO *et al.*, 2008 A polycomb group protein, PHF1, is involved in the response to DNA double-strand breaks in human cell. Nucleic Acids Research **36:** 2939-2947.

O'CONNELL, S., L. WANG, S. ROBERT, C. A. JONES, R. SAINT *et al.*, 2001 Polycomblike PHD fingers mediate conserved interaction with enhancer of zeste protein. J Biol Chem **276:** 43065-43073.

SZKLARCZYK, D., A. FRANCESCHINI, M. KUHN, M. SIMONOVIC, A. ROTH *et al.*, 2011 The STRING database in 2011: functional interaction networks of proteins, globally integrated and scored. Nucleic Acids Research **39:** D561-D568.

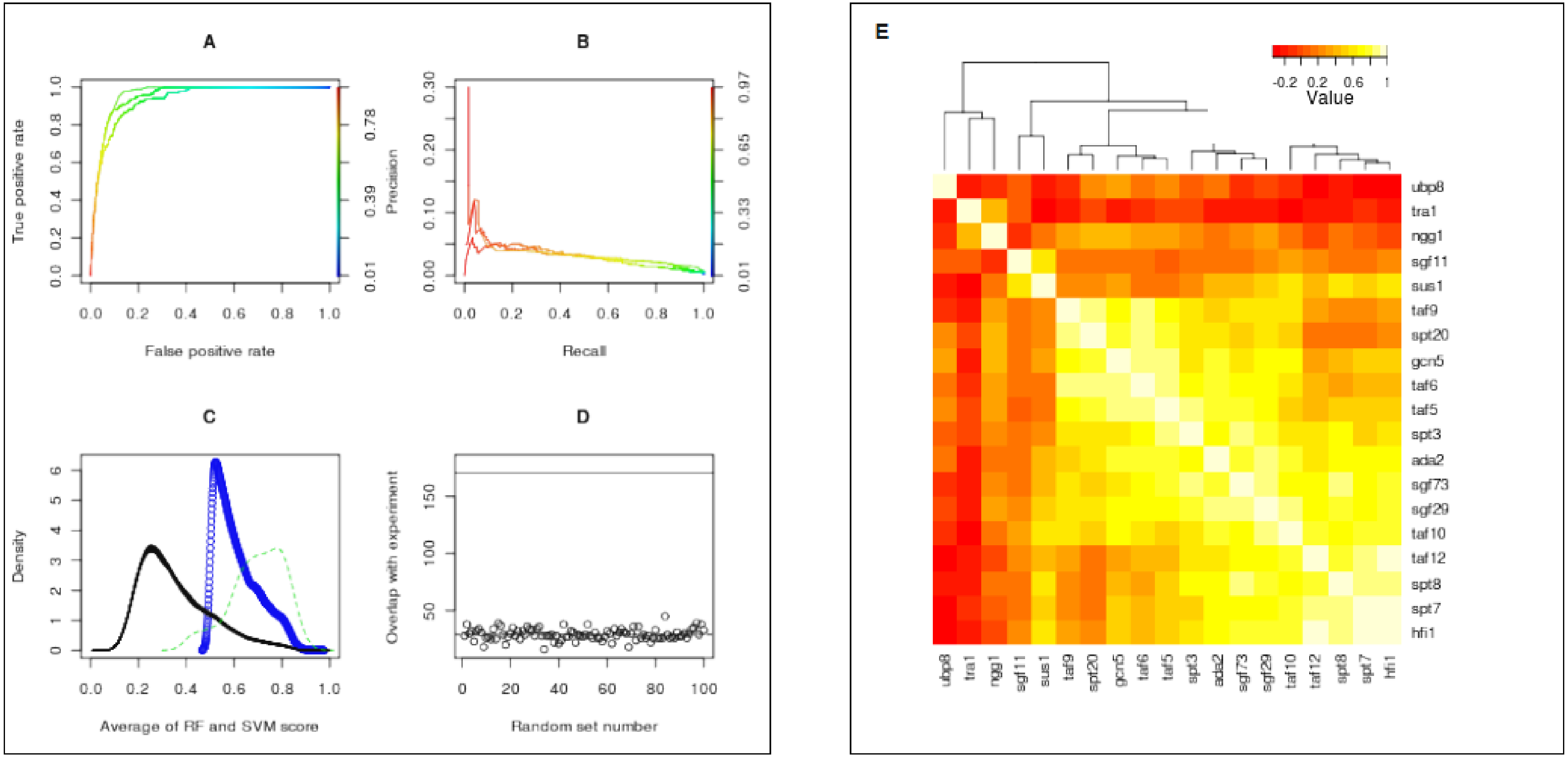

Figure S1 Analysis of predictions for fission yeast SAGA complex units. A) ROC curves for RF (upper curve), SVM (lower curve) and average scores (intermediate curves) obtained by comparing the predictions of within SAGA complex interactions with all interactions of SAGA units with other proteins. B) Corresponding precision-recall curves. C) Distributions of the predicted RF and SVM scores for interactions between SAGA units and other proteins (blue circles), distribution of scores of predictions of interactions amongst SAGA units (dashed green line), distribution of scores for predictions of interactions of 100 random sets of pairs including SAGA proteins (black lines). D) Overlap between the predicted interactors and the annotated interactions (solid line) and overlaps for each of the 100 random sets (the mean is shown by the dashed line). E) Heatmap showing clustering of the correlation matrix of predicted scores for interactions between different fission yeast SAGA complex subunits. The modules recapitulate the separate role of Ubp8 and Tra1, the close link of Spt7 and Hfi1 and sgf73 and sgf29, see main text (Helmlinger et al. 2011).

Helmlinger D, Marguerat S, Villen J, Swaney DL, Gygi SP, Bahler J, Winston F. 2011. EMBO J 30(14): 2843-2852.

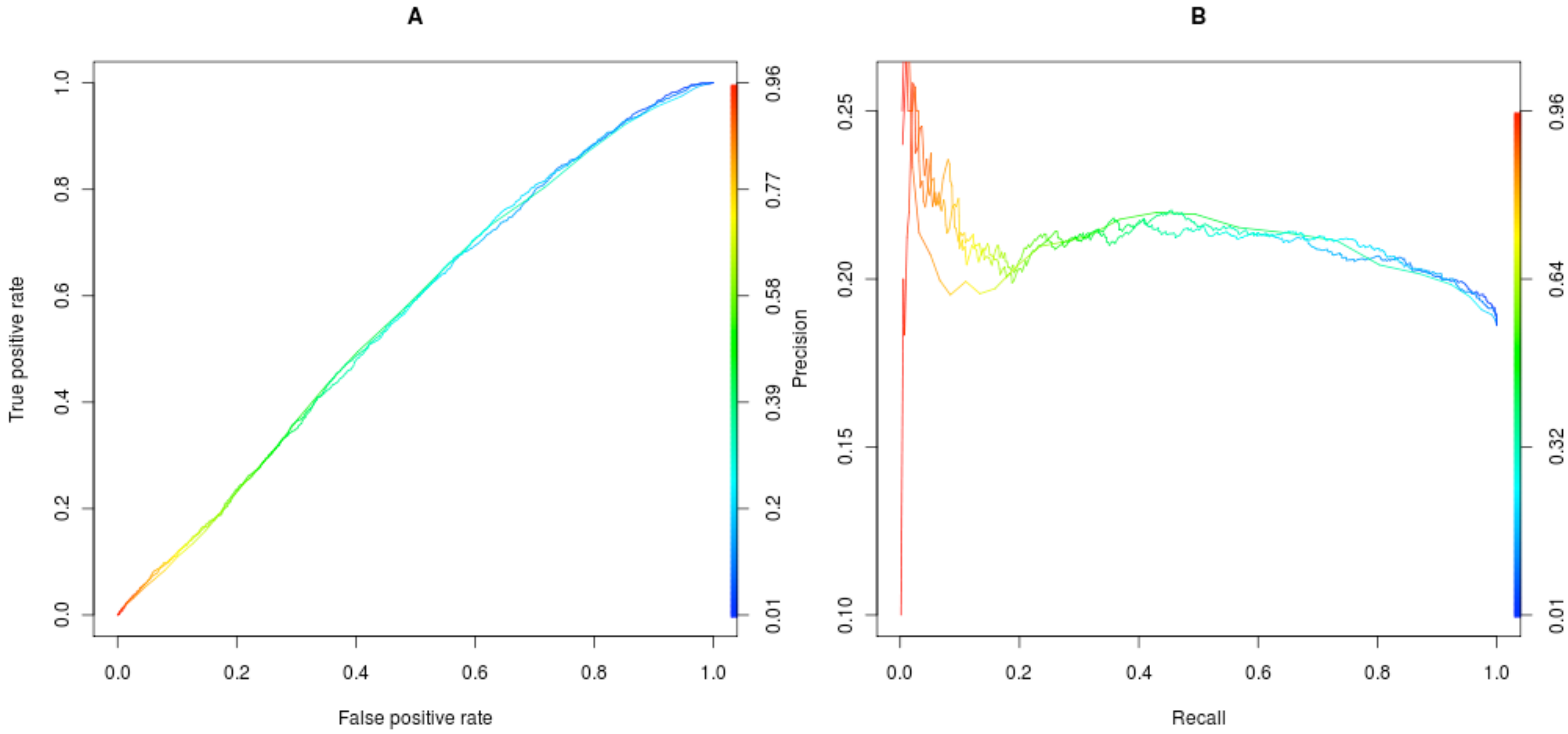

Figure S2 Comparison of SVM, RF and average SVM-RF predictions with the curated list of complexes in fission yeast (main text). A) ROC curve. B) Precision-recall curve. We simplistically assumed that interactions would happen between the all units of the annotated complexes and not between units annotated to different complexes. This leads to a set of 28,771 interactions and 1,374,879 non-interactions. The assumptions made mean that these non-interactions will contain some positives. Using a threshold of 0.5 for both SVM and RF, we confirm 14720 of the interactions and 1,191,506 of the non-interactions, with a precision of 0.07 and a FDR of 0.93. Raising the SVM threshold to 0.9 changes these values to 0.11 and 0.8, respectively.

You are looking for interactions amongst 25 proteins:

SPAC16A10.07C SPAC3A12.11C SPBC28F2.04C SPAC1782.09C SPCC962.02C SPAC2C4.14C SPBC428.13C SPAC664.07C SPAC17H9.19C SPAC664.01C SPAC17G8.10C SPBC6B1.10 SPAC13C5.02 SPBC1778.02 SPAC29A4.08C SPAC24H6.05 SPAC15A10.15 SPAC1834.06C SPAC227.07C SPBC19G7.06 SPBP23A10.07 SPBC4C3.05C SPCC622.16C SPBC244.01C SPCC663.11

(common names: **taz1, cwf2, cwf7, clp1, bir1, ppk11, mob1, rad9, cdt2, swi6, dma1, prp17, dre4, rap1, prp19, cdc25, sgo2, pmo25, pab1, mbx1, rpa2, nuc1, epe1, sid4, saf1**)

Predicted with **RF and SVM** above score **0.5** and showing a maximum of **100** hits.

View results in a table Download predictions

Interaction Network

Your query proteins are represented as red nodes in the network. The other nodes are predicted interactors and the link thickness is proportional to the confidence of the prediction (either SVM, RF or an avergae of both scores depending on your selection above).
The predictions that match known interactions (from BioGRID) are shown in colour as in the table. Use the controls to set the zoom level and centre the network.

Powered by Cytoscape Web

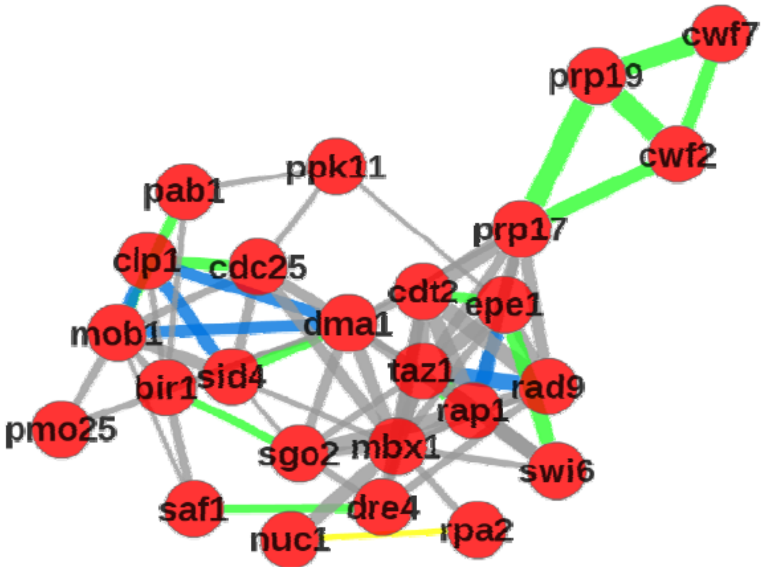

Rank	protein 1	protein 2	common name 1	common name 2	product 1	product 2	SVM score	RF score
[illegible]	SPAC16A10.07C	SPBC1778.02	taz1	rap1	human TRF ortholog Taz1	telomere binding protein Rap1	0.961604	0.806
[illegible]	SPAC17H9.19C	SPBC6B1.10	cdt2	prp17	WD repeat protein Cdt2	splicing factor Prp17	0.880434	0.687
[illegible]	SPBC19G7.06	SPBC4C3.05C	mbx1	nuc1	MADS-box transcription factor Mbx1	DNA-directed RNA polymerase I complex large subunit Nuc1	0.879543	0.568
[illegible]	SPAC29A4.08C	SPAC3A12.11C	prp19	cwf2	ubiquitin-protein ligase E4	RNA-binding protein Cwf2	0.872513	0.893
[illegible]	SPAC1782.09C	SPBC428.13C	clp1	mob1	Cdc14-related protein phosphatase Clp1/Flp1	Sid2-Mob1 kinase complex regulatory subunit Mob1	0.854687	0.57
[illegible]	SPBC244.01C	SPBC428.13C	sid4	mob1	SIN component scaffold protein Sid4	Sid2-Mob1 kinase complex regulatory subunit Mob1	0.847112	0.56
[illegible]	SPAC17G8.10C	SPBC19G7.06	dma1	mbx1	mitotic spindle checkpoint protein Dma1	MADS-box transcription factor Mbx1	0.845378	0.568
[illegible]	SPAC1782.09C	SPBC244.01C	clp1	sid4	Cdc14-related protein phosphatase Clp1/Flp1	SIN component scaffold protein Sid4	0.843502	0.574
[illegible]	SPAC29A4.08C	SPBC6B1.10	prp19	prp17	ubiquitin-protein ligase E4	splicing factor Prp17	0.842339	0.879
0	SPAC29A4.08C	SPBC28F2.04C	prp19	cwf7	ubiquitin-protein ligase E4	splicing factor Cwf7	0.842227	0.841
1	SPAC664.01C	SPBC1778.02	swi6	rap1	chromodomain protein Swi6	telomere binding protein Rap1	0.841489	0.637
2	SPAC17H9.19C	SPAC664.07C	cdt2	rad9	WD repeat protein Cdt2	checkpoint clamp complex protein Rad9	0.83628	0.769
3	SPAC664.01C	SPCC622.16C	swi6	epe1	chromodomain protein Swi6	Jmjc domain chromatin associated protein Epe1	0.836035	0.636
4	SPAC17H9.19C	SPBC1778.02	cdt2	rap1	WD repeat protein Cdt2	telomere binding protein Rap1	0.827786	0.745
5	SPAC15A10.15	SPBC1778.02	sgo2	rap1	inner centromere protein, shugoshin Sgo2	telomere binding protein Rap1	0.820281	0.633
6	SPAC16A10.07C	SPCC622.16C	taz1	epe1	human TRF ortholog Taz1	Jmjc domain chromatin associated protein Epe1	0.812203	0.674
7	SPAC16A10.07C	SPAC664.01C	taz1	swi6	human TRF ortholog Taz1	chromodomain protein Swi6	0.805673	0.673
8	SPAC1782.09C	SPAC17G8.10C	clp1	dma1	Cdc14-related protein phosphatase Clp1/Flp1	mitotic spindle checkpoint protein Dma1	0.802976	0.535
9	SPAC24H6.05	SPBC19G7.06	cdc25	mbx1	M phase inducer phosphatase Cdc25	MADS-box transcription factor Mbx1	0.79385	0.582
0	SPAC17H9.19C	SPBC19G7.06	cdt2	mbx1	WD repeat protein Cdt2	MADS-box transcription factor Mbx1	0.793258	0.572
1	SPBC1778.02	SPCC622.16C	rap1	epe1	telomere binding protein Rap1	Jmjc domain chromatin associated protein Epe1	0.786539	0.662
2	SPAC227.07C	SPBC428.13C	pab1	mob1	protein phosphatase regulatory subunit Pab1	Sid2-Mob1 kinase complex regulatory subunit Mob1	0.782145	0.511
3	SPAC16A10.07C	SPBC19G7.06	taz1	mbx1	human TRF ortholog Taz1	MADS-box transcription factor Mbx1	0.779527	0.567
4	SPAC24H6.05	SPBC244.01C	cdc25	sid4	M phase inducer phosphatase Cdc25	SIN component scaffold protein Sid4	0.778353	0.541
5	SPAC17G8.10C	SPBC428.13C	dma1	mob1	mitotic spindle checkpoint protein Dma1	Sid2-Mob1 kinase complex regulatory subunit Mob1	0.775738	0.557

Figure S3 Screenshot for predictions of all interactions of proteins which have new reported high-confidence interactions since 2010.

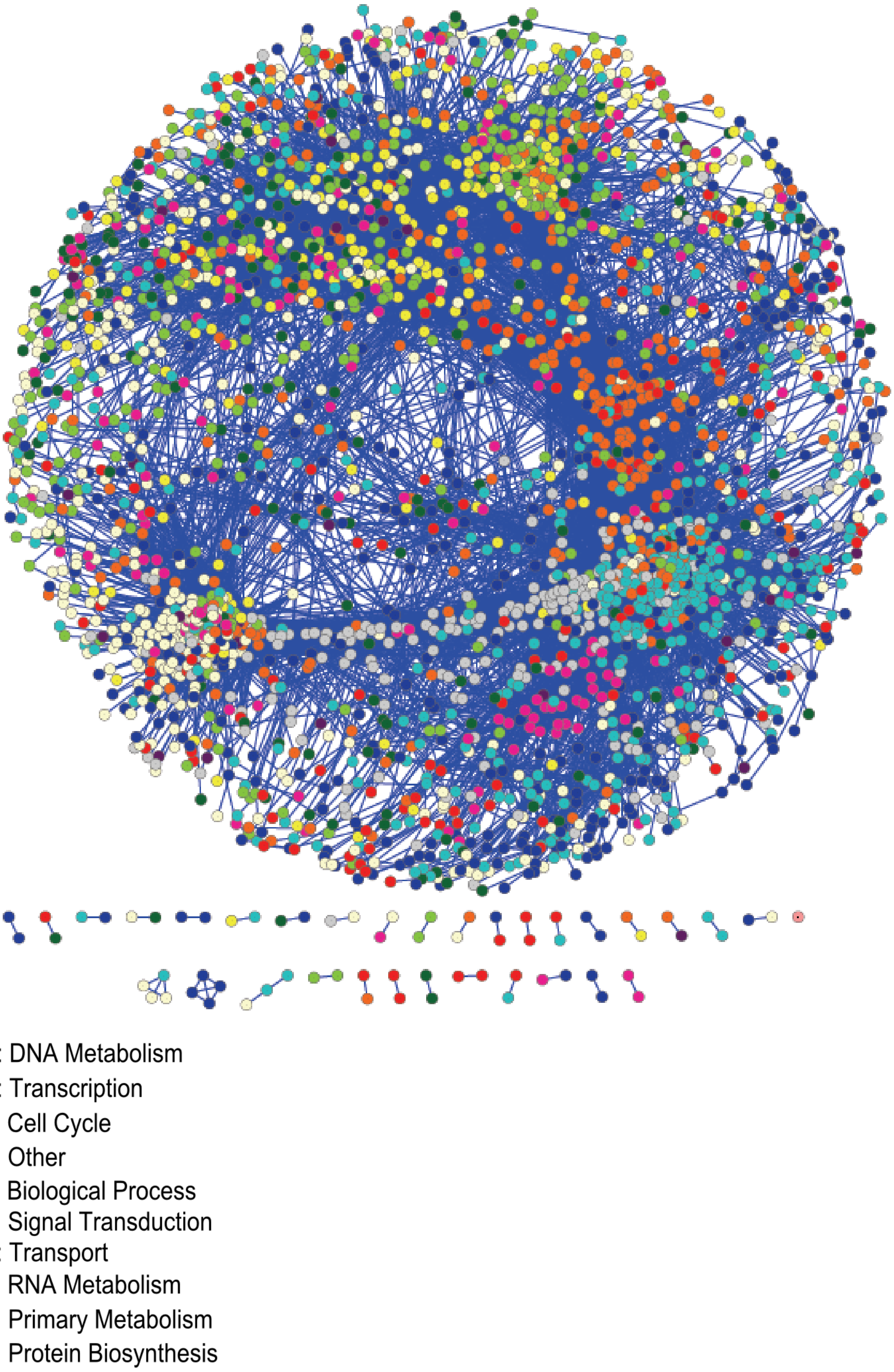

Figure S4 Interactome obtained by eliminating Gene Ontology features for the training. Most of the GO clusters can be recovered, showing redundancy within the features and implying that even proteins for which GO terms are not defined can be set in the correct neighbourhood.
