# Supplementary material for: Predicting the Fission Yeast Protein Interaction Network
Source: G3 (Bethesda). 2012 Apr 1;2(4):453–67. doi: 10.1534/g3.111.001560 (PMC3337474; doi:10.1534/g3.111.001560)
Supplement: Supporting Information [file supp_2.4.453_FigureS1.pdf]

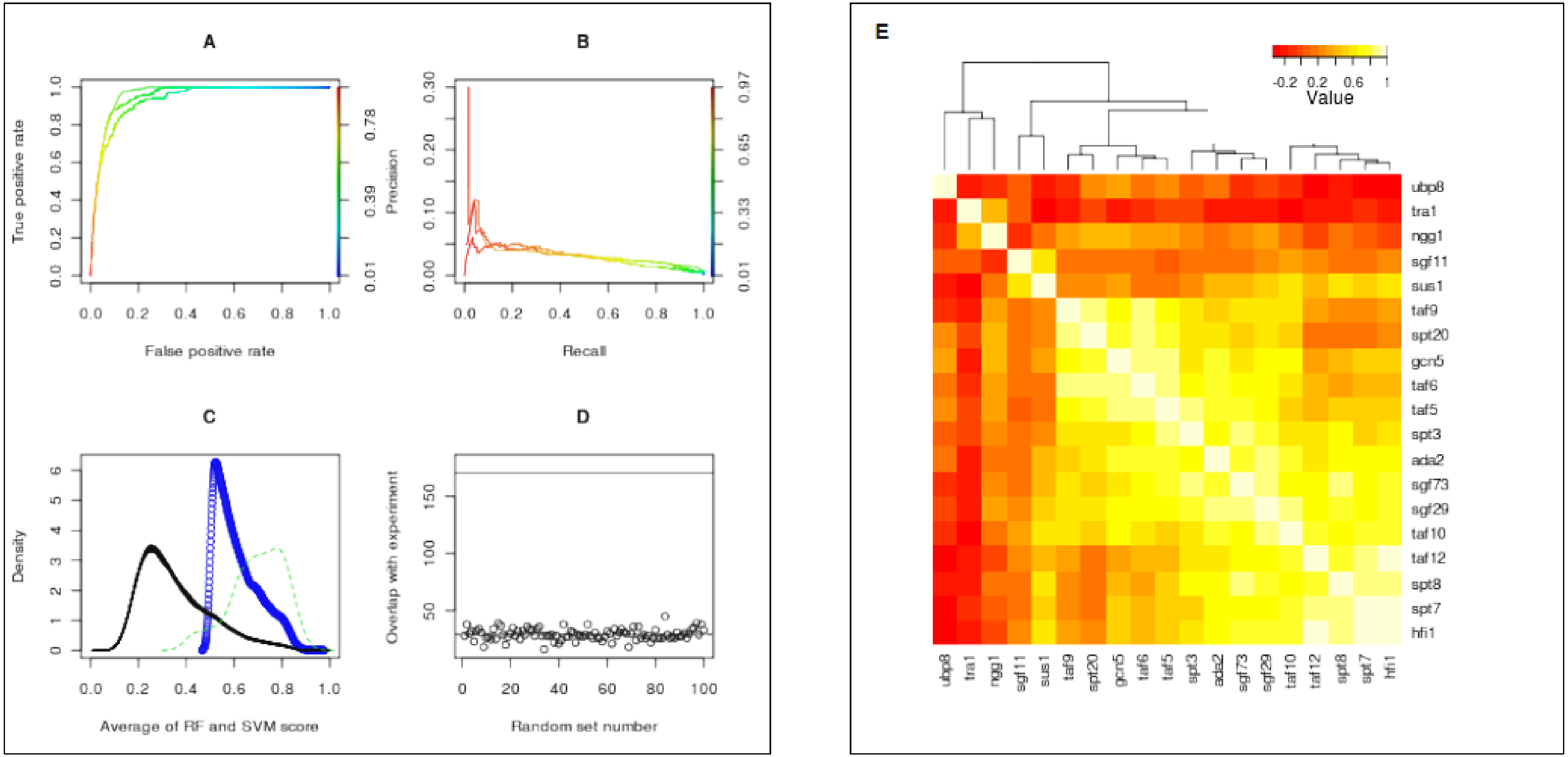

Figure S1 Analysis of predictions for fission yeast SAGA complex units. A) ROC curves for RF (upper curve), SVM (lower curve) and average scores (intermediate curves) obtained by comparing the predictions of within SAGA complex interactions with all interactions of SAGA units with other proteins. B) Corresponding precision-recall curves. C) Distributions of the predicted RF and SVM scores for interactions between SAGA units and other proteins (blue circles), distribution of scores of predictions of interactions amongst SAGA units (dashed green line), distribution of scores for predictions of interactions of 100 random sets of pairs including SAGA proteins (black lines). D) Overlap between the predicted interactors and the annotated interactions (solid line) and overlaps for each of the 100 random sets (the mean is shown by the dashed line). E) Heatmap showing clustering of the correlation matrix of predicted scores for interactions between different fission yeast SAGA complex subunits. The modules recapitulate the separate role of Ubp8 and Tra1, the close link of Spt7 and Hfi1 and sgf73 and sgf29, see main text (Helmlinger et al. 2011).

Helmlinger D, Marguerat S, Villen J, Swaney DL, Gygi SP, Bahler J, Winston F. 2011. EMBO J 30(14): 2843-2852.
