# Supplementary material for: Predicting the Fission Yeast Protein Interaction Network
Source: G3 (Bethesda). 2012 Apr 1;2(4):453–67. doi: 10.1534/g3.111.001560 (PMC3337474; doi:10.1534/g3.111.001560)
Supplement: Supporting Information [file supp_2.4.453_FigureS2.pdf]

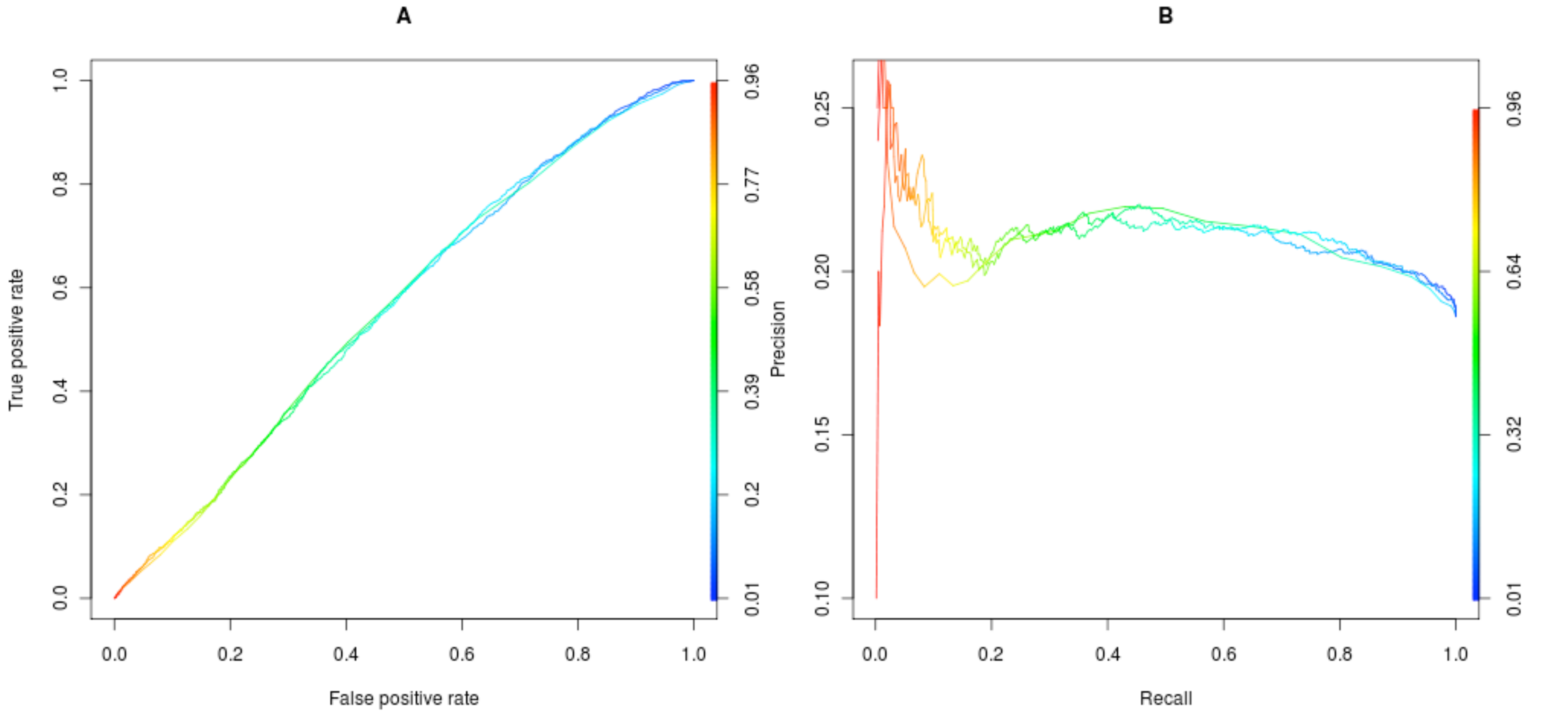

Figure S2 Comparison of SVM, RF and average SVM-RF predictions with the curated list of complexes in fission yeast (main text). A) ROC curve. B) Precision-recall curve. We simplistically assumed that interactions would happen between the all units of the annotated complexes and not between units annotated to different complexes. This leads to a set of 28,771 interactions and 1,374,879 non-interactions. The assumptions made mean that these non-interactions will contain some positives. Using a threshold of 0.5 for both SVM and RF, we confirm 14720 of the interactions and 1,191,506 of the non-interactions, with a precision of 0.07 and a FDR of 0.93. Raising the SVM threshold to 0.9 changes these values to 0.11 and 0.8, respectively.
