# Supplementary material for: Predicting the Fission Yeast Protein Interaction Network
Source: G3 (Bethesda). 2012 Apr 1;2(4):453–67. doi: 10.1534/g3.111.001560 (PMC3337474; doi:10.1534/g3.111.001560)
Supplement: Supporting Information [file supp_2.4.453_FigureS3.pdf]

You are looking for interactions amongst 25 proteins:

SPAC16A10.07C SPAC3A12.11C SPBC28F2.04C SPAC1782.09C SPCC962.02C SPAC2C4.14C SPBC428.13C SPAC664.07C SPAC17H9.19C SPAC664.01C SPAC17G8.10C SPBC6B1.10 SPAC13C5.02 SPBC1778.02 SPAC29A4.08C SPAC24H6.05 SPAC15A10.15 SPAC1834.06C SPAC227.07C SPBC19G7.06 SPBP23A10.07 SPBC4C3.05C SPCC622.16C SPBC244.01C SPCC663.11

(common names: **taz1, cwf2, cwf7, clp1, bir1, ppk11, mob1, rad9, cdt2, swi6, dma1, prp17, dre4, rap1, prp19, cdc25, sgo2, pmo25, pab1, mbx1, rpa2, nuc1, epe1, sid4, saf1**)

Predicted with **RF and SVM** above score **0.5** and showing a maximum of **100** hits.

View results in a table Download predictions

Interaction Network

Your query proteins are represented as red nodes in the network. The other nodes are predicted interactors and the link thickness is proportional to the confidence of the prediction (either SVM, RF or an avergae of both scores depending on your selection above).
The predictions that match known interactions (from BioGRID) are shown in colour as in the table. Use the controls to set the zoom level and centre the network.

Powered by Cytoscape Web

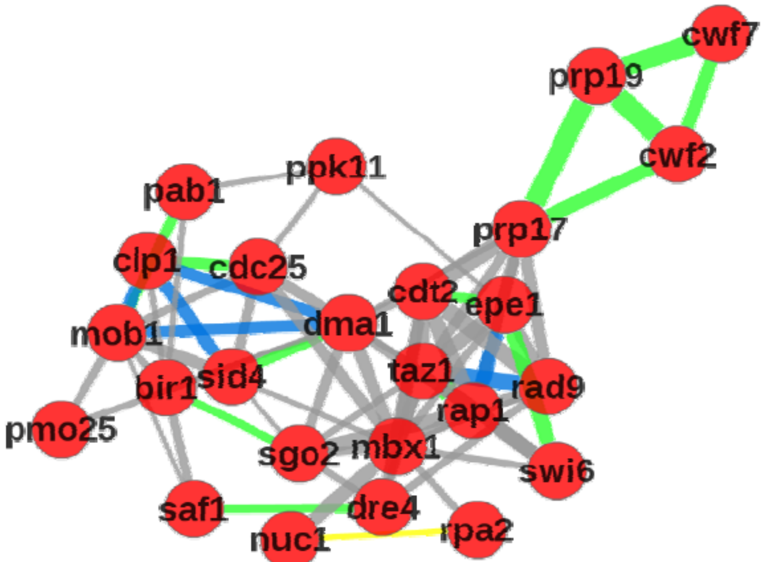

Rank	protein 1	protein 2	common name 1	common name 2	product 1	product 2	SVM score	RF score
[illegible]	SPAC16A10.07C	SPBC1778.02	taz1	rap1	human TRF ortholog Taz1	telomere binding protein Rap1	0.961604	0.806
[illegible]	SPAC17H9.19C	SPBC6B1.10	cdt2	prp17	WD repeat protein Cdt2	splicing factor Prp17	0.880434	0.687
[illegible]	SPBC19G7.06	SPBC4C3.05C	mbx1	nuc1	MADS-box transcription factor Mbx1	DNA-directed RNA polymerase I complex large subunit Nuc1	0.879543	0.568
[illegible]	SPAC29A4.08C	SPAC3A12.11C	prp19	cwf2	ubiquitin-protein ligase E4	RNA-binding protein Cwf2	0.872513	0.893
[illegible]	SPAC1782.09C	SPBC428.13C	clp1	mob1	Cdc14-related protein phosphatase Clp1/Flp1	Sid2-Mob1 kinase complex regulatory subunit Mob1	0.854687	0.57
[illegible]	SPBC244.01C	SPBC428.13C	sid4	mob1	SIN component scaffold protein Sid4	Sid2-Mob1 kinase complex regulatory subunit Mob1	0.847112	0.56
[illegible]	SPAC17G8.10C	SPBC19G7.06	dma1	mbx1	mitotic spindle checkpoint protein Dma1	MADS-box transcription factor Mbx1	0.845378	0.568
[illegible]	SPAC1782.09C	SPBC244.01C	clp1	sid4	Cdc14-related protein phosphatase Clp1/Flp1	SIN component scaffold protein Sid4	0.843502	0.574
[illegible]	SPAC29A4.08C	SPBC6B1.10	prp19	prp17	ubiquitin-protein ligase E4	splicing factor Prp17	0.842339	0.879
10	SPAC29A4.08C	SPBC28F2.04C	prp19	cwf7	ubiquitin-protein ligase E4	splicing factor Cwf7	0.842227	0.841
11	SPAC664.01C	SPBC1778.02	swi6	rap1	chromodomain protein Swi6	telomere binding protein Rap1	0.841489	0.637
12	SPAC17H9.19C	SPAC664.07C	cdt2	rad9	WD repeat protein Cdt2	checkpoint clamp complex protein Rad9	0.83628	0.769
13	SPAC664.01C	SPCC622.16C	swi6	epe1	chromodomain protein Swi6	Jmjc domain chromatin associated protein Epe1	0.836035	0.636
14	SPAC17H9.19C	SPBC1778.02	cdt2	rap1	WD repeat protein Cdt2	telomere binding protein Rap1	0.827786	0.745
15	SPAC15A10.15	SPBC1778.02	sgo2	rap1	inner centromere protein, shugoshin Sgo2	telomere binding protein Rap1	0.820281	0.633
16	SPAC16A10.07C	SPCC622.16C	taz1	epe1	human TRF ortholog Taz1	Jmjc domain chromatin associated protein Epe1	0.812203	0.674
17	SPAC16A10.07C	SPAC664.01C	taz1	swi6	human TRF ortholog Taz1	chromodomain protein Swi6	0.805673	0.673
18	SPAC1782.09C	SPAC17G8.10C	clp1	dma1	Cdc14-related protein phosphatase Clp1/Flp1	mitotic spindle checkpoint protein Dma1	0.802976	0.535
19	SPAC24H6.05	SPBC19G7.06	cdc25	mbx1	M phase inducer phosphatase Cdc25	MADS-box transcription factor Mbx1	0.79385	0.582
20	SPAC17H9.19C	SPBC19G7.06	cdt2	mbx1	WD repeat protein Cdt2	MADS-box transcription factor Mbx1	0.793268	0.572
21	SPBC1778.02	SPCC622.16C	rap1	epe1	telomere binding protein Rap1	Jmjc domain chromatin associated protein Epe1	0.786539	0.662
22	SPAC227.07C	SPBC428.13C	pab1	mob1	protein phosphatase regulatory subunit Pab1	Sid2-Mob1 kinase complex regulatory subunit Mob1	0.782145	0.511
23	SPAC16A10.07C	SPBC19G7.06	taz1	mbx1	human TRF ortholog Taz1	MADS-box transcription factor Mbx1	0.779527	0.567
24	SPAC24H6.05	SPBC244.01C	cdc25	sid4	M phase inducer phosphatase Cdc25	SIN component scaffold protein Sid4	0.778353	0.541
25	SPAC17G8.10C	SPBC428.13C	dma1	mob1	mitotic spindle checkpoint protein Dma1	Sid2-Mob1 kinase complex regulatory subunit Mob1	0.775738	0.557

Figure S3 Screenshot for predictions of all interactions of proteins which have new reported high-confidence interactions since 2010.
