# Supplementary material for: Predicting the Fission Yeast Protein Interaction Network
Source: G3 (Bethesda). 2012 Apr 1;2(4):453–67. doi: 10.1534/g3.111.001560 (PMC3337474; doi:10.1534/g3.111.001560)
Supplement: Supporting Information [file supp_2.4.453_FigureS4.pdf]

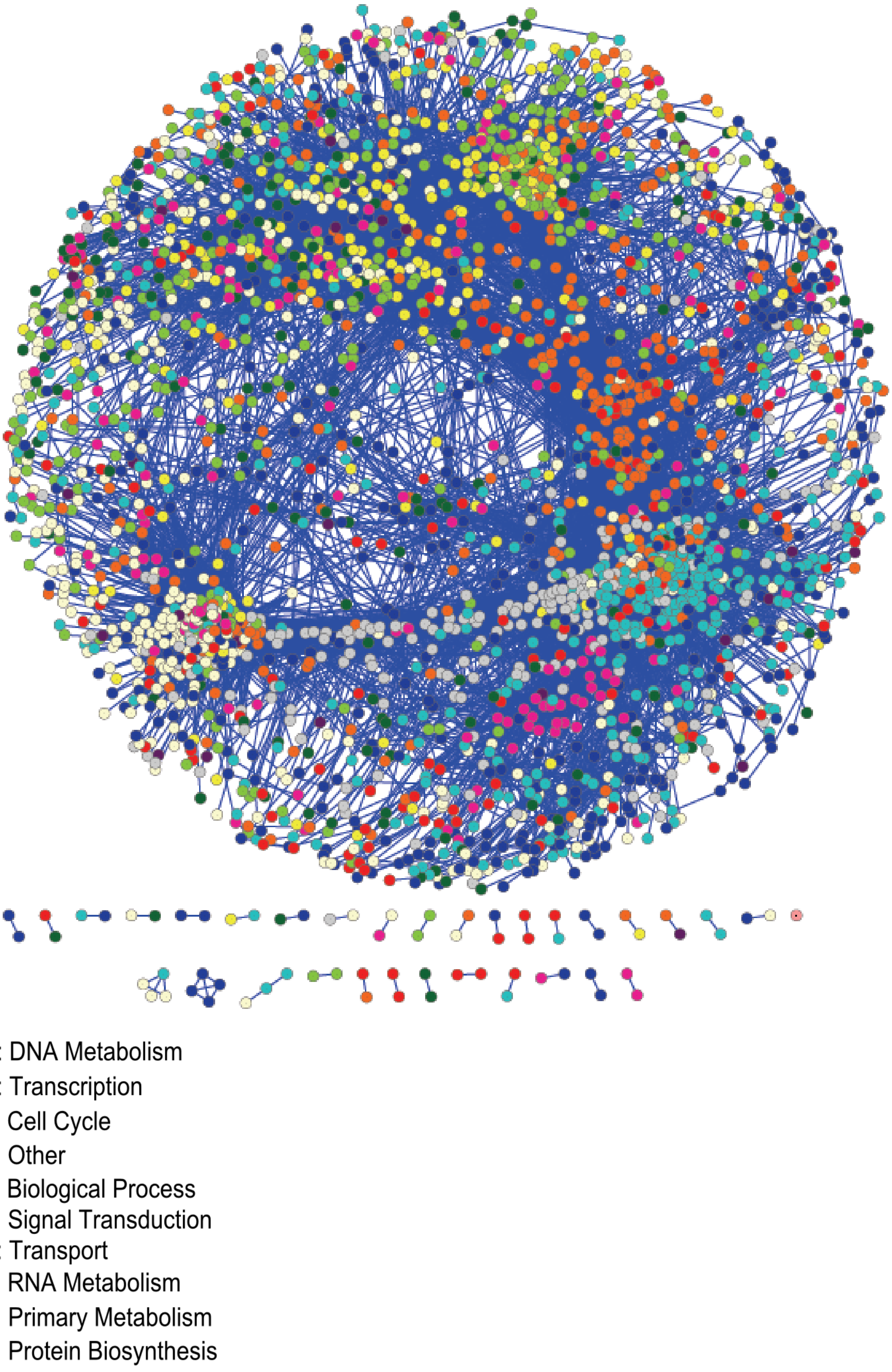

Figure S4 Interactome obtained by eliminating Gene Ontology features for the training. Most of the GO clusters can be recovered, showing redundancy within the features and implying that even proteins for which GO terms are not defined can be set in the correct neighbourhood.
