# Supplementary material for: Predicting the Fission Yeast Protein Interaction Network
Source: G3 (Bethesda). 2012 Apr 1;2(4):453–67. doi: 10.1534/g3.111.001560 (PMC3337474; doi:10.1534/g3.111.001560)
Supplement: Supporting Information [file supp_2.4.453_FileS9.pdf]

File S9

Phf1 is known to interact with Ezh2, orthologue to the fly Polycomb group protein Enhancer of zeste (O'CONNELL *et al.* 2001). In fly, Pcl (Polycomblike) is an orthologue of Phf1 known to interact with Enhancer of zeste, which itself interacts with orthologues of members of the SAGA complex (Pcaf, Ada2b, …). This makes a link between Phf1 and the fission yeast SAGA complex very plausible. In human, Phf1 is known to interact with Ku80 (related to pku80, which we also predict to associate with the SAGA complex) (HONG *et al.* 2008). Moreover, the fly homologue of Cdc17 (CG5602) is also predicted to indirectly interact with polycomb group proteins and a few of the SAGA orthologues in STRING (SZKLARCZYK *et al.* 2011). Information collected with the use of iHOP (http://www.ihop-net.org/).
