# Supplementary material for: Predicting the Fission Yeast Protein Interaction Network
Source: G3 (Bethesda). 2012 Apr 1;2(4):453–67. doi: 10.1534/g3.111.001560 (PMC3337474; doi:10.1534/g3.111.001560)
Supplement: Supporting Information [file supp_2.4.453_FileS10.pdf]

File S10

Our incomplete knowledge of the fission yeast interactome makes it impossible to know how many real interactions are within our negative sets. If we consider the expected size of the fission yeast interactome we can estimate how many true interactions we would expect in a specific number of random draws. For every 12.5 million pairs we expect around 60000 to be true, a ratio of 0.006. Hence, if we assume the network is equally dense in different regions, we expect 0.006 real interactions for every pair that we draw. Since we have ~32000 supposedly negative interactions in our negative test set, drawn at random, up to around 200 could be potentially true. If that were the case, our estimates of True Positives and False Positives would change drastically (given that the known true positives are only 204 adding 200 doubles this number) doubling the precision from 2% to 5%.This highlights how big the error margin on these estimates is in the absence of a completely known interactome.

HONG, Z., J. JIANG, L. LAN, S. NAKAJIMA, S. KANNO *et al.*, 2008 A polycomb group protein, PHF1, is involved in the response to DNA double-strand breaks in human cell. Nucleic Acids Research **36:** 2939-2947.

O'CONNELL, S., L. WANG, S. ROBERT, C. A. JONES, R. SAINT *et al.*, 2001 Polycomblike PHD fingers mediate conserved interaction with enhancer of zeste protein. J Biol Chem **276:** 43065-43073.

SZKLARCZYK, D., A. FRANCESCHINI, M. KUHN, M. SIMONOVIC, A. ROTH *et al.*, 2011 The STRING database in 2011: functional interaction networks of proteins, globally integrated and scored. Nucleic Acids Research **39:** D561-D568.
